# Getting to the root of HIV transmitted founder virus sequences

**DOI:** 10.1093/ve/veag025

**Published:** 2026-04-20

**Authors:** Bradley R Jones, Zabrina L Brumme, Eric Hunter, Jeffrey B Joy

**Affiliations:** Department of Mathematics, Simon Fraser University, Burnaby, Canada; BC Centre for Excellence in HIV/AIDS, Vancouver, Canada; Faculty of Health Science, Simon Fraser University, Burnaby, Canada; Emory Vaccine Center, Emory University, Atlanta, GA, United States; Department of Pathology & Laboratory Medicine, Emory University School of Medicine, Atlanta, GA, United States; BC Centre for Excellence in HIV/AIDS, Vancouver, Canada; Department of Medicine, University of British Columbia, Vancouver, Canada; Bioinformatics Program, University of British Columbia, Vancouver, Canada

**Keywords:** human immunodeficiency virus, persistent reservoir, ancestral sequence reconstruction, transmitted/founder virus, phylogenetic rooting

## Abstract

Determining the sequence of the transmitted founder virus, the virus that establishes infection in a new host, is critical for understanding early viral dynamics and evolution. Methods to estimate transmitted founder virus sequences using ancestral sequence reconstruction require either sequences collected early in infection or longitudinal samples that can capture the evolutionary history of the viral sequences, which can be challenging to collect. In human immunodeficiency virus infection, viral genomes are integrated into host cells which can persist, creating a proviral archive of the evolutionary history of the virus. We can potentially utilize these proviral sequences, which can be collected later during infection and while the individual is on therapy, to perform ancestral sequence reconstruction to estimate founder virus sequences. We analysed a previously described data set of 12 participants from Zambia who had human immunodeficiency virus sequences collected within months of infection and proviral sequences collected before and after suppressive therapy initiation. We investigated the accuracy of root placement and founder virus sequence reconstruction in these individuals from their proviral sequences using a variety of phylogenetic methods. We had limited success in reconstructing founder virus sequences across all ancestral sequence reconstruction and rooting methods. However, we observed lower error in founder virus sequence reconstruction with participants that had proviral sequences similar to their founder sequence. Our results highlight a need for new methods to be developed in order to effectively reconstruct founder virus sequences from proviral sequences.

## Introduction

The transmitted founder virus is the virus transmitted to a new host that establishes itself and ultimately leads to infection. In human immunodeficiency virus 1 (HIV-1) infection, typically only one viral lineage makes it past the transmission bottleneck to establish infection (Derdeyn et al. 2004, Keele et al. 2008, Joseph et al. 2015, Tully et al. 2016). Reconstructing the sequence of the transmitted founder virus can advance our understanding of the transmission bottleneck at the viral (Keele et al. 2008, Tully et al. 2016) and host levels (Lama and Planelles 2007, Keele and Estes 2011), assess within-host virus evolution (Shankarappa et al. 1999, Fischer et al. 2010), evaluate the presence of transmitted drug resistance mutations (Lingappa et al. 2021), measure the efficacy of preventive vaccines (Haynes et al. 2022, Landovitz et al. 2023), and investigate HIV transmission within a population (Hassan et al. 2017).

Reconstructing transmitted founder virus sequences is useful because it identifies the viral genotype that successfully crossed the transmission bottleneck and established productive infection. This can inform prevention of new HIV infections in at least three ways. First, founder virus sequences assist in defining viral features that are preferentially transmitted, a fact that is highly relevant to understanding mucosal transmission (Keele et al. 2008, Tully et al. 2016) and for design and evaluation of vaccines, broadly neutralizing antibodies, and other HIV prevention strategies (Haynes et al. 2022, Landovitz et al. 2023). Second, comparison of founder virus and subsequent within-host sequences provides a baseline for measuring early viral adaptation, including appearance or persistence of transmitted resistance associated variants (Lingappa et al. 2021). Third, if founder-like sequences could be inferred from samples collected long after infection, in scenarios where acute infection samples are rarely available this could expand the number of individuals contributing informative genomes to molecular epidemiology and phylodynamic studies. However, currently, whether chronic proviral sequence data retain enough information to support accurate founder virus sequence reconstruction remains unclear.

Historically, efforts to reconstruct HIV founder virus sequences required the collection of within-host HIV sequence datasets sampled during acute HIV infection, and ideally longitudinally thereafter (Keele et al. 2008, McCloskey et al. 2014). For ancestral sequence reconstruction of founder virus sequences, Keele et al. (2008) used the consensus sequence (CS) of early collected HIV sequences and McCloskey et al. (2014) used Bayesian time tree inference followed by maximum likelihood methods to reconstruct codons and indels with longitudinal HIV samples. But, identifying individuals with acute HIV infection and collecting such early or longitudinal sequence datasets is extremely challenging, limiting our understanding of founder virus sequences. By contrast, cohorts of individuals with chronic HIV infection are far more abundant, and could potentially be leveraged for founder virus reconstruction, if a method for extracting ancestral information from HIV sequences sampled years after infection can be developed.

Even when longitudinal within-host sequence datasets are available, determining the founder virus sequence in HIV infection can still be challenging because the virus undergoes rapid evolution with many selection sweeps (Shankarappa et al. 1999). As a result, even richly sampled longitudinal HIV RNA sequence datasets may not carry enough information to estimate ancestral sequences going back to the time of infection. Integrated HIV proviruses, by contrast, preserve some of this historic within-host evolutionary history and could potentially be leveraged in this regard. HIV, like all retroviruses, integrates its genome into that of the host cell as part of its replication cycle (Finzi et al. 1997, Mbonye and Karn 2024). While most infected cells will rapidly produce new HIV virions, resulting in the cell (and provirus) perishing (Finzi et al. 1997), a minority will remain largely transcriptionally dormant and persist long-term in what is called the HIV ‘persistent reservoir’ (Kreider and Bar 2022) or ‘latent reservoir’ (Mbonye and Karn 2024). These latent proviruses can persist for decades (Jones et al. 2018) and since they are not actively replicating they will undergo minimal evolution, making their sequence essentially indistinguishable from viral sequences circulating at the time of their integration. Thus, the persistent reservoir constitutes an archive of ancestral viral sequences that can extend back to the time of infection (Brooks et al. 2020), and therefore can potentially be used for ancestral sequence reconstruction. However, because proviruses are chronologically heterogeneous (in that their sampling date does not reflect their true chronological age), it is challenging to determine which sequences from a sample are older *versus* younger, which is essential if we are to correctly reconstruct the transmitted founder virus sequence.

Using phylogenetic methods, we can perform ancestral sequence reconstruction (ASR) to estimate founder virus sequences by associating these sequences with the root of the phylogeny. The simplest method of ancestral sequence reconstruction is parsimony, first developed by Camin and Sokal and Zuckerkandl and Pauling (Camin and Sokal 1965, Zuckerkandl and Pauling 1965), which aims to minimize the number of substitutions (or the cost of substitutions) on the phylogeny. Modern software that can perform parsimony include: the R package phangorn (Schliep 2011) and PAUP* (Swofford 2003). Another approach is to use likelihood methods. These involve using Felsenstein’s tree likelihood (Felsenstein 1981) to find the maximum likelihood of the sequence at the desired position (Schliep 2011, Ashkenazy et al. 2012, Minh et al. 2020) or to sample the sequence from a posterior distribution using Bayesian methods (Ronquist et al. 2012, Baele et al. 2025).

Here, we attempt to estimate founder virus sequences from 12 published within-host HIV datasets solely using provirus sequences. The datasets derive from participants of a Zambian cohort for whom plasma HIV RNA sequences were collected within weeks of infection, and proviral sequences were collected before and after suppressive therapy initiation. We first investigate the accuracy of root placement in proviral phylogenies using different rooting methods. Then using the CS of the plasma sequences as the ground truth for the founder virus sequence, we assess the effect of root position on our ability to estimate the sequence of the transmitted founder virus using a variety of phylogenetic methods ranging from parsimony to maximum likelihood and Bayesian analysis.

## Materials and methods

### Data curation

We curated 1130 sequences from 12 individuals living with HIV from the Zambia-Emory HIV Research Project (Brooks et al. 2020). Blood samples were collected: at seroconversion (SC), shortly before cART initiation (CC), ~1 year after cART initiation (1A), and for four individuals, and ~2 years after cART initiation (2A). HIV RNA sequences were defined from plasma virus from SC samples and HIV DNA sequences were obtained from peripheral blood mononuclear cells (PBMCs) from samples collected during cART. For five individuals, HIV DNA sequences were also defined from PBMCs from the samples collected shortly before cART initiation. In Brooks et al. (2020), plasma samples collected 1 year after SC and shortly before cART initiation were analysed, but these were not included in our analyses. See Brooks et al. (2020) for further details. Sequences were then trimmed to the *env* and *nef* regions for all further analyses.

A reference sequence data set of HIV subtype C *env* sequences from Zambia was curated from the Los Alamos National Laboratory (LANL) HIV Sequence Database (https://www.hiv.lanl.gov/) downloaded on 31 May 2023 by filtering Subtype (C), Genome region (Env CDS), and Sampling country (ZM). This resulted in 4112 sequences. A second data set of HIV *nef* sequences from the LANL HIV Sequence Database (https://www.hiv.lanl.gov/) was downloaded and curated on 17 January 2024 by filtering Subtype (C), Genome region (Nef CDS), and Sampling country (ZM) resulting in 2389 sequences.

For each reference sequence data set, we aligned the sequences using MAFFT v7.526 (Katoh and Standley 2013) with local pair alignment. We reduced each data set to distinct sequences using a custom R script and then removed sequences from participants in our study from the reference data set. For each individual in the data sets, we reduced their sequences to one CS of sequences from their earliest collection time using the R package seqinr. Patients lacking collection time information were reduced to a single CS each and sequences lacking patient ID were left as is. This resulted in reference-participant alignments with 179 sequences (*env)* and 473 sequences (*nef*).

### Quality control and sequence sorting

We aligned each participant’s data set from the Zambia-Emory HIV Research Project using MAFFT (Katoh and Standley 2013) with local pair alignment. We reduced each participant data set to distinct sequences using a custom R script. We sorted the sequences by collection date using a custom R script. CSs from SC time points were computed using a custom R script. We screened the sequences for hypermutation using the hypermut.R script from the GitHub repository (https://github.com/brj1/brjtools) that mimics the behaviour of the software Hypermut (Lapp et al. 2025) using the CSs as the reference sequence. At this point, we separated the proviral sequences and realigned with MAFFT and removed columns that contained at least 50% missing data. We censored sequences that displayed evidence of recombination using RDP v4.101 (Martin et al. 2015) with a *P*-value <.05 after Bonferroni correction for at least three recombination detection methods (3Seq, BootScan, Chimaera, Geneconv, MaxChi, RDP, and Siscan). Finally, columns that now contained at least 50% missing data were removed.

Each CS was aligned to its participant’s alignment using the ‘add --keeplength’ command in MAFFT. The CSs resulting from these alignments were used to represent a proxy for the founder virus sequence for each participant.

### Phylogenetic inference

To each reference CS data set from LANL, we added the proviral sequences from each participant and aligned with MAFFT (Katoh and Standley 2013) using local pair alignment and then columns that contained at least 50% missing data were removed. We removed recombinant sequences using RDP4 as above. We call the resulting alignment the reference-participant alignment. A maximum likelihood phylogenetic tree was inferred from this alignment using IQ-TREE v2.4.0 (Minh et al. 2020). We call this the reference-participant phylogeny. We selected the best-fitting nucleotide substitution model using IQ-TREE’s extended model selection with default parameters.

Subsequently, to recover phylogenies for each participant, we inferred a maximum likelihood tree with IQ-TREE from each participant’s alignment of proviral sequences. We selected the best-fitting nucleotide substitution model using IQ-TREE’s extended model selection with default parameters. The best-fitting substitution models for each participant are shown in Tables S1 and S2.

### Rooting

**Figure 1:**
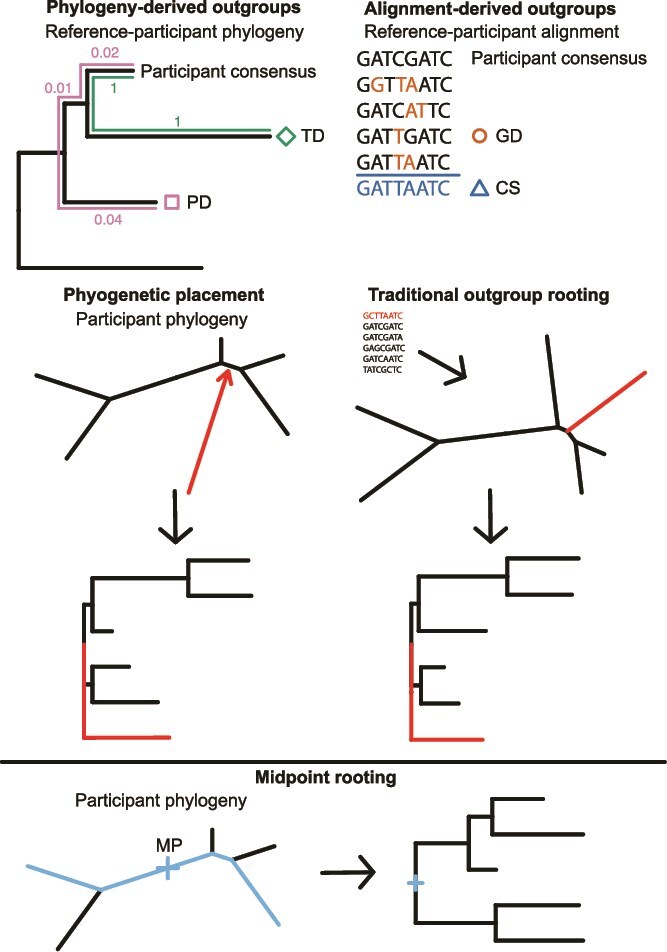
Diagram of outgroup selection and rooting methodologies. TD selects the sequence in the reference-participant phylogeny that is the fewest number of branches from our participant CS. PD selects the sequence in the reference-participant phylogeny that has the shortest path (in substitutions per site) to our participant consensus. GD selects the sequence in the reference-participant alignment that has the fewest differences (scaled by a Markov model) from our participant CS. CS constructs the consensus of the reference-participant alignment. With outgroup selected, the outgroup sequence (TD, PD, GD, or CS) is either inserted into the maximum likelihood position in the participant phylogeny (phylogenetic placement) or is added to the participant alignment and a new phylogeny is inferred (traditional outgroup rooting). MP selects the position in the participant phylogeny that is at the midpoint of a longest path in the phylogeny. See the Materials and Methods section for more details.

We selected outgroup sequences for each participant data set in one of five ways: (1) genetic distance (GD), (2) phylogenetic distance (PD), (3) topological distance (TD), (4) CS, and (5) transmitted founder virus (TF). (1) For GD, we selected the sequence in reference-participant alignment that had the smallest TN93 GD (computed with the ‘dist.dna’ function in the R package ape; Tamura and Nei 1993; Paradis and Schliep 2019) to our participant’s consensus proviral sequence. (2) For PD, we computed the PD in the reference-participant phylogeny using the ‘cophenetic’ function in ape and chose the sequence with lowest PD to our participant’s consensus proviral sequence. (3) For TD, first we collapsed branches in the reference-participant with length less than 1 × 10^−4^ subs. Per site, and then we selected the sequence in the reference-participant phylogeny that minimizes the quantity, $D(s)$:


$$ D(s)={d}_T\left(s,p\right)+\frac{d_P\left(s,p\right)}{N} $$

where $p$ is our participant sequence, ${d}_T\left(s,p\right)$ is the TD between $s$ and $p$, ${d}_P\left(s,p\right)$ is the PD between $s$ and $p$, $N$ is the number of tips in the tree, and $s$ runs over the sequences in the reference-participant phylogeny. Minimizing this quantity essentially finds the closest sequence topologically using PD to break ties. This is because ${d}_T\left(s,p\right)\gg \frac{d_P\left(s,p\right)}{N}$. We computed this value using the ‘cophenetic’ function in ape. For CS, we computed the CS of the alignment of the reference data set using a custom R script. Finally, for TF, we simply used the CS we are using as a proxy for the founder virus sequence as our outgroup sequence. This outgroup was considered to evaluate the performance of each ancestral sequence reconstruction method independent of outgroup. See Fig. 1 for a diagram of the outgroup selection methods.

For the parsimony, indel-aware parsimony, empirical Bayes, and both maximum likelihood methods that are explained in the following section we used the same outgroup rooted trees. For each participant and each of their associated outgroup sequences, we added the outgroup sequence to the participant’s alignment and then aligned with MAFFT (Katoh and Standley 2013). Subsequently, phylogenetic placement was performed with IQ-TREE (Minh et al. 2020) by inferring a tree from the alignment including the outgroup sequence, but constraining the topology of the proviral sequences to the phylogeny inferred in the previous section. The root position was then determined in the original tree using a custom R script. We also created outgroup rooted trees by inferring a tree in IQ-TREE from the alignment including the outgroup sequence without constraining the tree topology for the participant proviral sequences.

Additionally, we rooted the trees with midpoint rooting (MP) using the R package phangorn (Schliep 2011); we denote this method with MP. This finds the unique point in the phylogeny that is the midpoint of a longest path in the phylogeny. These midpoint rooted trees were used with the parsimony, indel-aware parsimony, empirical Bayes, and both maximum likelihood methods of the following section.

### Ancestral sequence reconstruction

We compared six methods of ASR as outlined in more detail below. Each method was performed on each of the rooted phylogenies generated in the previous section.

#### Parsimony

Maximum parsimony ASR was performed in the R package phangorn (Schliep 2011) using the MPF method with the best-fitting available substitution model as computed by IQ-TREE (see Tables S1 and S2).

#### Indel-aware parsimony

Indel-aware parsimony ASR was performed in indelMAP (Ighault et al. 2024) with the best-fitting available substitution model as computed by IQ-TREE (see Tables S1 and S2) and default parameters for gap costs.

#### Empirical Bayes

Empirical Bayes ASR was performed in the R package phangorn (Schliep 2011) with the best-fitting available substitution model as computed by IQ-TREE (see Tables S1 and S2).

#### Maximum likelihood

Maximum likelihood ASR was performed in the R package phangorn (Schliep 2011) with the best-fitting available substitution model as computed by IQ-TREE (See Tables S1 and S2). Maximum likelihood ASR was also performed in IQ-TREE (Minh et al. 2020) using the best-fitting substitution as computed by IQ-TREE (see Tables S1 and S2). For IQ-TREE, a placeholder tip had to be added to the tree at the root position and sequence with all ‘?’ had to be added to the alignment in order to specify the sequence to reconstruct.

#### Markov chain Monte Carlo

Bayesian methods using Markov chain Monte Carlo were performed in MrBayes v3.2.7 (Ronquist et al. 2012) with the best-fitting available substitution model as computed by IQ-TREE (see Tables S1 and S2). For each participant and outgroup, we aligned the outgroup sequence to the participant’s alignment using the ‘--add --keeplength’ to preserve the participant’s alignment structure and length. With each of these alignments, we ran two parallel runs of four chains with 33 335 000 generations each sampling every 5000 generations to simultaneously infer the tree topology and ancestral sequence between the participant and outgroup sequences. Convergence was assessed by standard deviation of split frequencies below 0.05 and effective sample sizes of all parameters above 200. We combined the samples of the two runs after removing 25% burn-in. We considered the estimated ancestral sequence to be the mode sampled nucleotide at each base.

#### Using the outgroup sequence to inform the ancestral sequence reconstruction

We reperformed each ASR method by including the outgroup sequence in our alignment. When rooting each phylogeny, we retained the tip representing the outgroup sequence. We estimated the ancestral sequences at the node separating the outgroup from the participant sequences using the alignments created for the Markov chain Monte Carlo analyses.

### Statistical methods

We evaluated error in ancestral sequence reconstruction methods by computing the number of nucleotide bases that differ between the estimated sequence and the proxy founder virus sequence. This quantity was then divided by the number of variable bases in the alignment of the proviral sequence and proxy transmitted founder virus. Kruskal-Wallis tests and analysis of variance were performed in R. The R package coda (Plummer et al. 2006) was used to compute effective sample sizes. Multiple sequence alignment input and output were performed in R with the package sequinr (Charif and Lobry 2007) and tree manipulation was performed with the R packages phytools (Revell 2024), tidytree (Yu 2022), and treeio (Wang et al. 2020). Plots were created in R using ggplot (Wickham 2016) and ggtree (Yu et al. 2017). We also used the R package tidyverse (Wickham et al. 2019) in our R scripts. A full breakdown of the pipelines in this work, from alignment cleaning to ancestral sequence reconstruction, is shown in Fig. S1.

## Results

Our ultimate aim is to assess the potential to estimate the transmitted founder virus sequence of a person living with HIV solely from proviral HIV sequences. To do this, we performed the following steps detailed in the Methods section: (1) computed a proxy for the transmitted founder virus from the consensus of an early time point, (2) chose candidate outgroup sequences from a database of publicly available sequences, (3) rooted a proviral phylogeny using a variety of methods, (4) reconstructed the sequence of the root of our phylogeny using a variety of ancestral sequence reconstruction methods, and (5) compared our estimated sequence to our proxy transmitted founder virus sequence to assess error.

### Intrahost proviral data sets with ‘known’ transmitted founder virus

To properly evaluate the accuracy in estimating transmitted founder virus sequences, we require a data set where a reliable estimate of the actual founder virus sequence is known. We opted to use a previously published data set of HIV sequences from 12 individuals living with HIV from Zambia whose HIV diversity was consistent with acquisition of a single founder virus (Brooks et al. 2020). These participants were followed prior to infection and each have a negative HIV test a median 87.5 days [interquartile range: 36.25–92 days] to their initial positive HIV test result (SC). Upon diagnosis, each participant’s plasma was sampled and individual HIV sequences were amplified using single-genome methods. We use the consensus of each participant’s earliest collected sequences as a proxy for their founder virus sequence (we call this sequence TF). This is similar to previous studies (Keele et al. 2008, McCloskey et al. 2014). Each participant’s proviral HIV diversity was also assessed using single-genome amplification methods at up to three time points: CC: a few months prior to therapy initiation, 1A: during suppressive therapy, and 2A: later during suppressive therapy. See Brooks et al. (2020) for more details.

The HIV region sequenced covered the *vpu*, *env*, and *nef* genes. We truncated the sequences to include either the *env* or *nef* region and performed subsequent analyses with each region separately. This was done to simplify our task of recovering the founder sequence because the *env* and *nef* regions exhibit varied fitness pressure and within-host evolution that make it difficult to infer a multigene phylogeny. The *env* gene encodes the viral envelope protein (Prabakaran et al. 2007), and therefore mutations in *env* play a significant role in the virus’ ability to evade host immune responses (Prabakaran et al. 2007). The *nef* gene encodes the viral accessory protein Nef which promotes HIV replication and spread, in part through immune evasion functions (Staudt et al. 2020). *Nef* is less variable then *env*, but still accrues enough mutations within-host to study its evolution (Jones et al. 2018). After data cleaning, we obtained a median 28.5 [range: 8–43] (*env*) and 32 [range: 14–51] (*nef*) unique sequences per participant. A further breakdown of the number of sequences for each participant is given in Tables S3 and S4.

### Determining viable outgroup sequences

The primary method to determine phylogenetic root position is to use outgroup rooting (OGR). This is where we include a more distantly related taxon or taxa (called the outgroup) in our phylogeny and then select the intersection between the outgroup and the rest of phylogeny as the root (Wheeler 1990). The outgroup sequences can either be included in the initial tree inference (Wheeler 1990) or can be added to the phylogeny with phylogenetic placement (Matsen et al. 2010). This latter method is also called the evolutionary placement algorithm (Czech et al. 2019) and is often used to assess the validity of an outgroup root placement as it provides a likelihood score (Hubert et al. 2014, Liede-Schumman et al. 2020). It is important to select an appropriate outgroup sequence that is ancestrally divergent, but not too distantly related. This is to ensure that the outgroup root is indeed ancestral and to avoid long branch attraction (Wheeler 1990, Graham et al. 2002).

**Figure 2:**
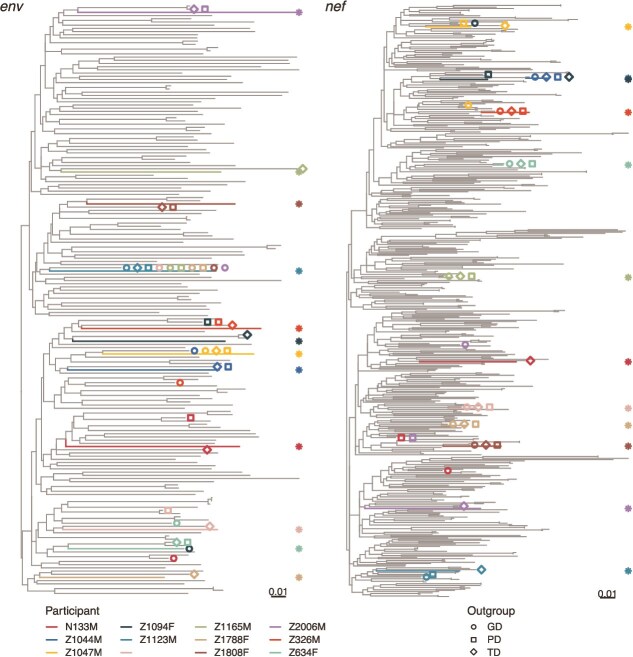
Maximum likelihood phylogenies of reference and participant data (reference-participant phylogenies). Coloured lines (and asterisks) indicate participant proviral CSs. Open symbols in matching colours indicate the sequences chosen as the outgroup for each participant. GD: Genetic distance root, PD: Phylogenetic distance root, TD: Topological distance root. Phylogenies were midpoint rooted for visualization.

We curated HIV *env* and *nef* sequences from Zambia from the LANL HIV Sequence Database to create reference sequence databases for each gene (see Materials and Methods section). We then added the CS of each of our participant’s proviral sequences to these databases and inferred a phylogenetic tree (the reference-participant phylogeny) from this reference-participant alignment. We used the participants’ CSs instead of including all proviral sequences in order to reduce the complexity of inferring the phylogenetic tree by using fewer sequences and to prevent within-host evolution from biasing reconstruction of the phylogenetic tree*.* We selected candidate sequences for OGR using one of three notions of closeness by finding the sequence in our reference-participant data set/phylogeny that has the smallest GD, PD, or TD to our participant consensus. GD counts the number of different nucleotides between the participant CS and candidate sequence (scaled by TN93 GD; Tamura and Nei 1993); this metric does not require inferring a large phylogenetic tree and thus can be computed quite quickly. PD measures the length of the path in the reference-participant phylogeny between the participant CS and candidate sequence in substitutions per site; this estimates the amount of evolution between each sequence and accounts for reverse mutations that are masked when computing GD. TD counts the number of branches on the path in the reference-participant phylogeny between the participant CS and candidate sequence; this somewhat represents the sequence with the fewest evolutionary events from our participant’s CS. See Fig. 1 for a diagram illustrating these methods. Figure 2 shows the *env* and *nef* phylogenetic trees of the reference sequences and participant proviral CS with candidate outgroup sequences highlighted. The GD outgroups were mean 0.114 (*env)* and 0.0414 (*nef*) subs. Per site from their participant consensus, the PD outgroups were mean 0.188 (*env*) and 0.0562 (*nef*) subs. per site from their participant consensus, and the TD outgroups were mean 2.5 (*env*) and 2.42 (*nef*) branches from their participant consensus. All participants’ outgroup sequences were chosen from the LANL sequences except for participant Z1094’s *env* outgroup for the GD metric, where participant Z634F’s consensus was chosen. Note that some methods chose the same outgroup sequence including across different participants. For example, the same sequence was chosen ten times among participants and methods in *env* (Fig. 2) and each method chose the same sequence for seven participants in *nef* (Fig. 2). The closest sequence by GD was sometimes in a very different place on the tree than the participant sequence, which is due to GD not completely aligning with PD. As an alternative to finding a closest sequence to an outgroup, we also considered the CSs of the *env* and *nef* reference sequence database as an outgroup sequence (CS). This CS approximates the most common ancestor of all the sequences and should serve as a viable outgroup. Like GD, it also does not require inferring a large phylogenetic tree and thus can be computed quickly.

### Rooting phylogenies of within-host proviral sequences is difficult

**Figure 3:**
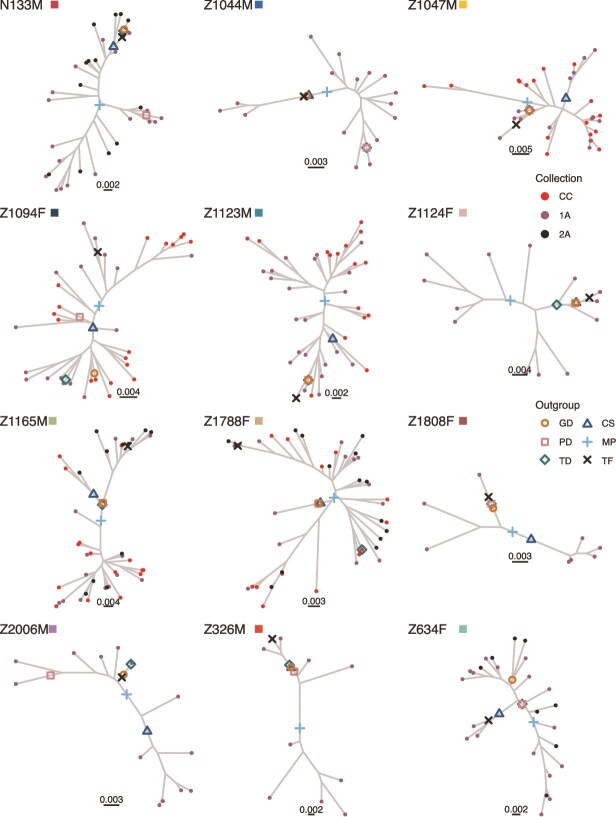
Proviral HIV *env* phylogenies with root placement. Open symbols correspond to root placement using different outgroup sequences (GD: Genetic distance root, PD: Phylogenetic distance root, TD: topological distance root, CS: Consensus sequence root). + Symbol corresponds to midpoint rooting. X symbol corresponds to root placement using the proxy transmitted/founder virus as outgroup. Tip symbols correspond to sampling time.

**Figure 4:**
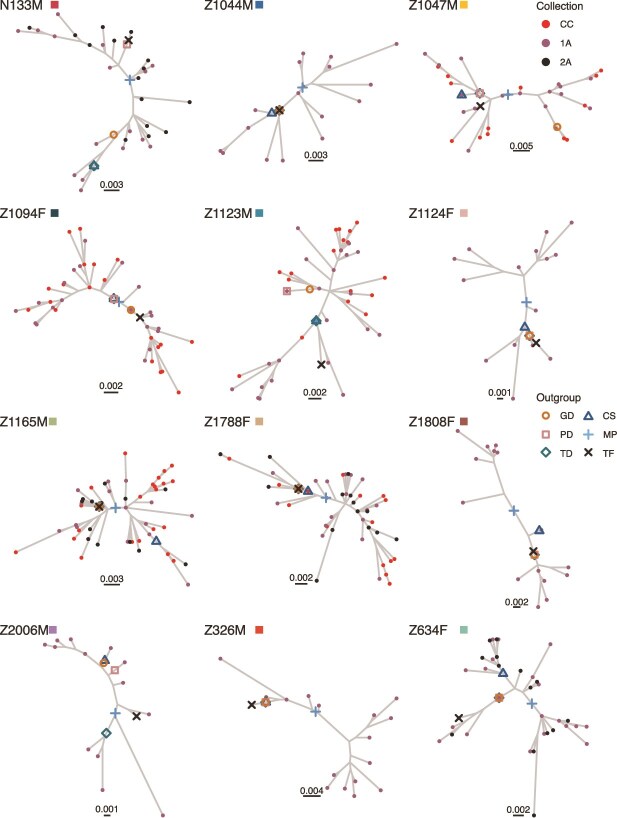
Proviral HIV *nef* phylogenies with root placement. Open symbols correspond to root placement using different outgroup sequences (GD: Genetic distance root, PD: Phylogenetic distance root, TD: Topological distance root, CS: Consensus sequence root). + Symbol corresponds to midpoint rooting. X symbol corresponds to root placement using the proxy transmitted/founder virus as outgroup. Tip symbols correspond to sampling time.

For each participant, we inferred a phylogeny from their proviral sequences using maximum likelihood methods in IQ-TREE (Minh et al. 2020). Next, we rooted the phylogenies with phylogenetic placement in IQ-TREE using each of the four outgroup sequences identified in the last section (GD, PD, TD, and CS) as well as the proxy transmitted/founder sequence (TF). Here, the TF sequence represents our best approximation of the actual root position in the tree that indicates the most recent common ancestor (MRCA) of our proviral sequences. In addition, we midpoint rooted the phylogenies (MP), which selects the position in the tree at the middle of a longest path. We considered MP as an alternative to outgroup grouping because (1) it doesn’t require data outside the participant and (2) it can be computed quickly. However, MP assumes that the sequences (or at least the most divergent sequences) have had same amount of evolution from the root (i.e. the sequences satisfy the molecular clock hypothesis) (Kinene et al. 2016), which is not generally the case in proviral data sets (Brodin et al. 2016, Jones et al. 2018). See Fig. 1 for an illustration of all our rooting methods. Figures 3 and 4 show each participant’s within-host phylogeny, where the root placement using each outgroup sequence is shown with open symbols, the midpoint is shown with a $+$, and the root placement using the proxy TF sequence is shown with an X. In each participant, the root positions selected varied considerably between method/outgroup. Consider the four statistics for each participant: ${t}_{avg}$, the average number of branches in the path between root positions in a participant, ${t}_{max}$, the maximum number of branches in the path between root positions in a participant, ${p}_{avg}$, the average length of the path in substitutions per site between root positions in a participant, and ${p}_{max}$, the maximum length of the path in substitutions per site between root positions in a participant. Among all participants the median ${t}_{avg}$ was 2.85 [IQR: 1.70–4.95], the median ${t}_{max}$ was 6 [IQR: 3.5–8], the median ${p}_{avg}$ was 8.70 × 10^−3^ [IQR: 8.06 × 10^−3^ to 1.19 × 10^−2^] subs. per site, and the median ${p}_{max}$ was 1.87 × 10^−2^ [IQR: 1.36 × 10^−2^ to 1.99 × 10^−2^] subs. per site. Nevertheless, in 8 of the 12 participants at least one method/outgroup provided the same or similar root position as TF (e.g. in *nef*, Z10788F and Z1665M in Fig. 4 all the outgroups inferred the same root position). However, some for participants the root positions were different for all outgroup sequences (see, e.g. *env* Z1094F in Fig. 3).

We assessed the error in the root placements by comparing the distance (topological (number of branches in the path between roots) or phylogenetic (length of the path between roots in substitutions per site)) from the inferred root position to the root position using the TF sequence as outgroup (see Tables S5 and S6). Overall, GD outgroups had lower error in *env* (mean TD: 3.67, mean PD: 1.20 × 10^−2^ subs. per site) and PD outgroups had lower error in *nef* (mean TD: 1.92, mean PD: 4.43 × 10^−3^ subs. per site), with higher accuracy in *nef*. However, no single outgroup selection method yielded an overall significant advantage as judged by a Kruskal-Wallis test (*P*-value: .559 (*env*), 0.167 (*nef*)).

Additionally, we performed OGR for each participant by inferring a phylogeny with IQ-TREE (Minh et al. 2020) by including the outgroup in the initial tree inference, which is a more traditional method of OGR. We did this for each outgroup (we did not redo MP). We chose to use phylogenetic placement initially because it removes any biases that could arise in the tree topology due to between host evolution. We cannot assess the error in root placement for this form of OGR by comparing the distances between root positions because different tree topologies are inferred for each outgroup. We instead used the Euclidean distance of the vectors of root-to-tip distances (see Tables S7 and S8) as our error metric. There was no significant advantage in outgroup selection as judged by a Kruskal-Wallis test (*P*-value: .635 (*env*), 0.9839 (*nef*)). There was also no significant difference between error of root position between phylogenetic placement and traditional OGR (paired *t*-test *P*-value: .253 (*env*), 0.950 (*nef*)). Here error is in terms of Euclidean distance of the vectors of root-to-tip distances.

### Correct root placement is essential but not sufficient for accurate ancestral sequence reconstruction

**Figure 5:**
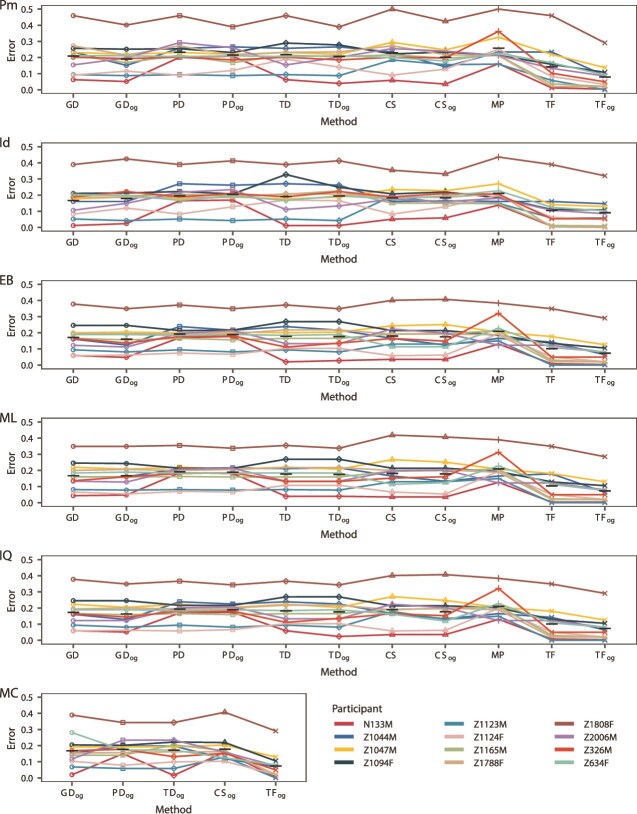
Error of ancestral sequence reconstruction methods of *env*. Error is number of incorrect bases divided by the number of variable sites (see description in the Materials and Methods section). Colour corresponds to participant and lines link replicates from the same participant. Black horizontal bars indicate the mean error for each outgroup. Pm: parsimony with phangorn; ID: parsimony with indelMAP, EB: empirical Bayes with phangorn, ML: maximum likelihood with phangorn, IQ: maximum likelihood with IQ-TREE, MC: Markov chain Monte Carlo with MrBayes. GD: genetic distance root, PD: phylogenetic distance root, TD: topological distance root, CS: consensus sequence root, MP: midpoint rooting, TF: founder sequence root; og: ancestral reconstructions that included the outgroup sequence.

**Figure 6:**
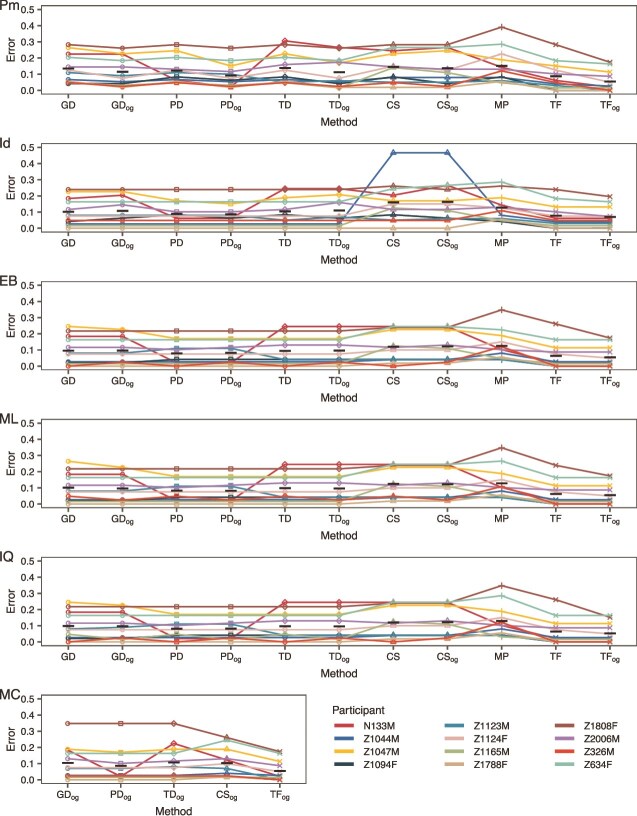
Error of ancestral sequence reconstruction methods of *nef*. Error is number of incorrect bases divided by the number of variable sites (see description in the Materials and Methods section). Colour corresponds to participant and lines link replicates from the same participant. Black horizontal bars indicate the mean error for each outgroup. Pm: parsimony with phangorn; ID: parsimony with indelMAP, EB: empirical Bayes with phangorn, ML: maximum likelihood with phangorn, IQ: maximum likelihood with IQ-TREE, MC: Markov chain Monte Carlo with MrBayes. GD: genetic distance root, PD: phylogenetic distance root, TD: topological distance root, CS: consensus sequence root, MP: midpoint rooting, TF: founder sequence root; og: ancestral reconstructions that included the outgroup sequence.

Next, we attempted to reconstruct the founder virus sequence for each participant using our rooted trees. We began by estimating the founder sequences from rooted phylogenies that did not include the outgroup as a tip with parsimony (with and without indel awareness), empirical Bayes, and maximum likelihood (with phangorn and IQ-TREE). The resulting error (the number of incorrect bases as compared to the proxy founder virus sequence (TF) divided by the number of variable sites) for each ASR method, outgroup method, and participant are shown in Figs 5 and 6. There was little difference in the error across different ASR methods or outgroup sequences using analysis of variance (ANOVA; *P*-values: .104 (outgroup, *env*), 0.025 (method, *env*); 0.030 (outgroup, *nef*), 0.154 (method, *nef*)). Next, we tried including the outgroup sequence in the tree. This allows the outgroup sequence to inform the ancestral sequence reconstruction. There was a significant improvement in error when including the outgroup (paired *t*-test *P*-value: <.001 (*env*), <0.001 (*nef*)), though, this is not true overall as, for example, empirical Bayes method performed better in *nef* without including the outgroup (see Fig. 6). We also included a Bayesian method (with MrBayes; Ronquist et al. 2012) which simultaneously infers the outgroup root position and founder virus sequence (see Figs 5 and 6), which performed comparably. Finally, we tried ancestral sequence reconstruction using phylogenies that were outgroup rooted by including the outgroup when inferring the phylogeny (the traditional method of OGR). This produced higher error than using phylogenies with roots selected by phylogenetic placement (paired *t*-test *P*-value: .001 (*env*), 0.414 (*nef*)). See Figs S2 and S3. Overall, the most accurate method was empirical Bayes using the GD outgroup *via* phylogenetic placement and including the outgroup in the ancestral sequence reconstruction for *env* (mean error: 0.158) and empirical Bayes using the PD outgroup *via* phylogenetic placement and not including the outgroup in the ancestral sequence reconstruction for *nef* (mean error: 0.078).

To better assess the utility of each method, we performed each ASR method using the proxy founder virus sequence (TF) as the outgroup (see Figs 5 and 6). This removes the effect that rooting has on each ASR method and can serve as a sort of null model. Unsurprisingly, error for all methods/data sets was lower using the TF as outgroup over the other outgroups (paired *t*-test all outgroups *P*-value: <.001), with some participants’ datasets approaching zero error regardless of ancestral reconstruction method used (e.g. N133M in *env* and Z1788F in *nef*, see Figs 5 and 6). However, the error for many data sets could still not be brought below 0.01. In fact, for participant Z1808F this error was very pronounced (see Figs 5 and 6). The reason for this is presumably because the TF sequence for this participant was placed on a very long branch, but the ASR methods estimate the sequence at the intersection of this branch and the rest of the tree, which could have many substitutions (see Fig. S4). In Tables S9 and S10, we give the founder sequence terminal branch length for all participants. Additionally, participants that included a sequence similar to the founder sequence had higher accuracy when using non-TF outgroups. We confirmed this with a correlation test between error and the minimum TN93 GD (Tamura and Nei 1993) between the founder sequence and the proviral sequences in each participant (Pearson *ρ*: 0.586, *P*-value: <.001 (*env*); Pearson *ρ*: 0.662, *P*-value: <.001 (*nef*)).

The largest variability in the error to reconstruct founder virus sequences came from the variability in the participant data sets. In particular the number of sequences (Pearson *ρ*: −0.272, *P*-value: <.001 (*env*); Pearson *ρ*: −0.288, *P*-value: <.001 (*nef*))/number of collection time points (Pearson *ρ*: −0.133, *P*-value: .001 (*env*); Pearson *ρ*: −0.241, *P*-value: <.001 (*nef*)) had a significant impact on the overall error.

To further explore this, we subsampled time points from our two participants with three proviral collection time points, Z1165M and Z1788F, and reperformed our analyses (including data cleaning, phylogenetic tree reconstruction, and ancestral sequence reconstruction but not reselecting outgroup sequences as this involves inferring a large phylogenetic tree in each replicate). In Z1165M, only including the CC time point (which was collected prior to cART initiation) resulted in higher error (see Figs S5 and S6). This behaviour was not observed in Z1788F, where there did not appear to be any pattern in *env* (see Fig. S7) and the Parsimony method had higher error when time points were removed while all other methods produced the same accuracy in *nef* except when using the CS outgroup or MP (see Fig. S8). In fact, removing time points always increased the error when MP in both participants. Overall, removing time points sometimes resulted in lower error across methods for both participants in *env* (see Figs S5 and S7). Z1165M (*nef*) and Z1788F (*env* and *nef*) had sequences that were identical to their founder virus sequence (disregarding indels). In Z1165M *nef*, this sequence was not present in the CC time point, which had higher error when only that time point was included. In Z1788F *nef*, all time points contained a sequence identical to the founder. In Z1165M, there was a significant correlation between minimum TN93 GD (Tamura and Nei 1993) to founder sequence and error in founder reconstruction (Pearson *ρ*: 0.755, *P*-value: <.001 (*env*); Pearson *ρ*: 0.748, *P*-value: <.001 (*nef*)). This highlights the importance of having similar sequences to the founder sequence in the proviral data set for obtaining accurate ancestral sequence reconstructions. The identical sequence of Z1788F *env* was in the CC time point, but it was removed in the CC + 2A data set because it was flagged as a recombinant and this data set had higher error when using the TF as the outgroup; however, the data sets that did not have the CC time point still produced similar error to the other data sets and there was no significant correlation between minimum TN93 GD (Tamura and Nei 1993) to founder sequence and error in founder reconstruction in Z1788F *env* (Pearson *ρ*: 0.059, *P*-value: .280).

There was no significant correlation between median collection time of proviral sequences and error between *nef* and *env* participant data sets (Pearson *ρ*: 0.053, *P*-value: .201 (*env*); Pearson *ρ*: −0.079, *P*-value: .056 (*nef*)), suggesting that sampling time may not be a factor for accurate founder sequence reconstruction using proviral sequences. Finally, we observed a significantly higher error in founder sequence reconstruction in our female participants (*n* = 5) over our male participants (*n* = 7) (*t*-test *P*-value: <.001 (*env*), .005 (*nef*)). It is possible that this difference in founder reconstruction capability could be due to the differences in the transmission route (and thus barrier) between male and female individuals (Joseph et al. 2015) or differences in immune pressure on the virus between sexes (Atfield and Ziegler 2016). However, it could also be an artefact of our data set. For example, in our data, there was a significant relationship between sex and minimum TN93 GD (Tamura and Nei 1993) to founder virus sequence, with male participants having more similar proviral sequences to their founder virus sequence (*t*-test *P*-value: <.01 (*env* and *nef*)). Prior to analysis, we removed hypermutated and recombinant sequences from our data sets, since they would misinform our phylogenetic inference. However, removing these sequences deprives us of the evolutionary information these sequences contain. To investigate this we compared the error in ASR to the proportion of sequences removed. This could only be done for *env* as no evidence of hypermutation or recombination was observed in *nef* (See Tables S3 and S4). There was a significant correlation between the number of sequences removed and error both as a proportion of sequences removed (Pearson *ρ*: 0.654, *P*-value: <.001) and the total number of sequence removed (Pearson *ρ*: 0.381, *P*-value: <.001). This correlation was still present when discretized by type of removed sequence (hypermutant proportion Pearson *ρ*: 0.195, *P*-value: <.001; hypermutant total Pearson *ρ*: 0.090, *P*-value: .030; recombinant proportion Pearson *ρ*: 0.596, *P*-value: <.001; recombinant total Pearson *ρ*: 0.349, *P*-value: <.001). This means that when more sequences were removed there was higher error in ASR. Recombination potentially plays more of a role in the participants that had more detected recombinants and removing these sequences biased our phylogenetic and ancestral sequence reconstructions.

## Discussion

Overall, we found that using proviral sequences collected a median of 3.86 years (range 2.32–6.70 years) following estimated HIV infection to reconstruct the sequences of transmitted founder virus in within-host HIV phylogenies resulted in poor accuracy. Various reasons underlie this poor accuracy in our ancestral sequence reconstruction. One is poor root placement. In some participants, our estimated root was up to 10 branching events away from the true root position (see Tables S5 and S6). In fact, this was true for all outgroups for participant Z1788F in *env* (see Fig. 3). Another reason is that the ASR methods infer the sequence at the intersection of the founder sequence branch and the tree. In some cases, however, the founder virus sequence was placed on a long branch and thus had substantially more substitutions than the sequence estimated by the ancestral reconstruction methods, which is at the intersection of the founder virus sequence branch and the rest of the tree. Finally, many founder virus sequences had indels (specifically missing bases; see Tables S11 and S12) which could not be estimated by our methods (except for the indel method).

A genetically/phylogenetically distant founder virus sequence was the main factor contributing to error in ancestral sequence reconstruction. This was most pronounced in participant Z1808F whose most genetically similar proviral sequence to the proxy founder sequence had a TN93 GD (Tamura and Nei 1993) of 1.71 × 10^−2^ subs. per site in *env* and 1.79 × 10^−2^ subs. per site in *nef*. Compare this to the maximum GD between proviral sequences (2.82 × 10^−2^ subs. per site (*env*), 3.68 × 10^−2^ subs. per site (*nef*)). This led to Z1808F having higher error than other participants (see Figs 4 and 5). In a previous study (Brooks et al. 2020), we estimated the integration dates of the proviral sequences and found that Z10808F’s proviral sequences were composed of younger viruses. This means that the MRCA of these sequences may only date to sometime in the middle of infection and reconstructing the sequence at the time of transmission is impossible.

We were able to obtain better root placement and higher accuracy in estimating founder virus sequences with *nef* sequences over *env* sequences. This is presumably due to *nef* being more mutationally stable and less subject to fitness pressures (Cuevas et al. 2015, Zanini et al. 2017).

One factor complicating our ancestral sequence reconstruction is HIV recombination. HIV virions contain two copies of HIV RNA. If two different virions infect the same cell, then one copy of each virion’s RNA can be packaged in the resulting virion and when this new virion infects a cell, recombination can occur when the RNA is reverse transcribed due to template switching (Rhodes et al. 2005). The presence of recombination introduces the possibility of multiple ancestry complicating phylogenetic and ancestral sequence reconstruction. In our study, we removed sequences that showed evidence of recombination. This removes the evolutionary information contained in these sequences possibly reducing power and introducing biases. Indeed, we had higher error when more recombinant sequences were detected and removed.

Similarly, hypermutation caused by the APOBEC3G gene introduces guanine to adenine mutations in the HIV proviral genome, which in turn complicates phylogenetic reconstruction by artificially grouping hypermutated sequences together (Kijak et al. 2008, Shahid et al. 2025). Because of this, we removed sequences with evidence of hypermutation. However, these hypermutated sequences may contain evolutionary information in non-hypermutated sites. A recent study (Shahid et al. 2025) explored using hypermutated sequences, but masking hypermutated sites, to assess the effect on estimating proviral integration dates. They found comparable results between removing hypermutated sequences and masking hypermutated sites.

Along with mutations, over time, HIV lineages accrue insertions and deletions, together called indels (Winters and Merigan 2005, Merendez-Arias et al. 2006, Wood et al. 2009). The evolutionary history of indels is difficult to reconstruct due to the changing sequence lengths, but indels play an important evolutionary role (Wood et al. 2009). In our study, we looked at one ASR method that incorporates indels, indelMAP (Ighault et al. 2024), but we only considered indels that preserve the overall alignment width, in particular indels that increase the ancestral sequence length. Furthermore, we found that indelMAP did not improve our accuracy in reconstructing the founder virus sequence over the other methods tested.

Even though our early samples used as a proxy for the founder virus were collected within a few months of infection, this sequence may not represent the true founder virus but rather a descendant of it that did not arise until a number of genetic bottlenecks had already occurred. During sexual HIV transmission, the transmitted founder virus must be present in the donor’s genital fluids (e.g. cervicovaginal mucus, semen, or rectal secretions) and then be transmitted to the recipient’s genital tract (Joseph et al. 2015, Kariuki et al. 2017). Viral genetic compartmentalization in the genital tract limits the within-host HIV variants that can transmitted (Joseph et al. 2015; Kariuki et al. 2017). HIV must then establish itself in mucosal surfaces and gut-associated lymphatic tissue of the recipient (Lama and Planelles 2007, Keele and Estes 2011, Kariuki et al. 2017), which favours viruses that are capable of using the C-C chemokine receptor type 5 coreceptor for viral entry (Lama and Planelles 2007, Keele and Estes 2011) and involves selection bias for more transmissible variants (Carlson et al. 2014). Following high levels of HIV replication during the acute phase of infection (Pilcher et al. 2004), viral loads decline sharply as host adaptive immune responses target the virus (Pilcher et al. 2004). Together, these factors (donor genital tract, recipient mucosal barrier and early host immune responses) create strong genetic bottlenecks that allow only one (or few) variants to persist (Pilcher et al. 2004, Keele et al. 2008, Joseph et al. 2015, Tully et al. 2016, Kariuki et al. 2017).

To further explore the effects of using sequences collected a few months after infection, we performed an analysis using publicly available sequences. We downloaded all HIV *env* sequences from the LANL HIV Sequence Database (https://www.hiv.lanl.gov/) that were collected within 100 days of the estimated date of infection (EDI) and filtered the sequences to come from individuals living with HIV for which there was at least one sample collected within 10 days of the EDI and at least one sample collected within 30–100 days of the EDI (34 individuals and 1216 sequences total). For each individual, we compared the CS of their earliest time point (collected within 10 days of their EDI) to the consensus of each of their time points collected 30–100 days after their EDI. There was a mean of 99.7% sequence identity between the consensus of the earliest time point and the consensus of each sample collected 30–100 days after EDI with the lowest sequence identity being 94.4%. This suggests that our choice of proxy founder was probably not that different from the actual founder virus sequence.

Our participants all likely acquired HIV through heterosexual transmission. Alternative transmission routes may exhibit different bottlenecks, evolutionary pressures, and dynamics for the founder viruses. For example, transmission from injection drug use and sexual transmission between men who have sex with men have a higher propensity to produce multiple founder variants than heterosexual transmission (Baxter et al. 2023). It is possible that ASR methods will perform differently when the route of transmission is different. We may have observed this in our study, since we had variable error between male and female participants and in heterosexual transmission the transmission barrier varies between sexes (Joseph et al. 2015).

Here, we analysed proviral sequences sequenced using single genome amplification (SGA), which only sequences a single isolate from a sample. However, next generation sequencing techniques allow detection of multiple sequences in a sample revealing diversity within a sample and generating more sequences per time point. Using these technologies may improve our ability to infer founder virus sequences because they may be able to capture sequences similar to the founder virus that are below the limit of detection for SGA. It may also be worthwhile to explore simulated data sets to thoroughly assess the effects of sample size, evolution, and participant attributes on ancestral sequence reconstruction.

In our study, we used *env* and *nef* sequences, but ancestral sequence reconstruction could also be attempted for other regions of the HIV genome or the whole genome. This would be imperative when investigating drug resistance mutations, since these mutations normally appear in regions which are targeted by antiretrovirals including *pol* and *gag* (Nasti et al. 2023). Whole genome reconstruction would be especially challenging because recombination would be a greater issue.

When determining candidate outgroup sequences, we used CSs of both the reference participant sequences and our study participant sequences. This was done to decrease the computation time needed and remove any within-host effects from the phylogenetic inference. However, using CSs removes data from our amylases that could inform our results. Due to HIV’s high level of within-host evolution and selective sweeps (Shankarappa et al. 1999), an individual’s HIV CS may represent a variant present in the middle of infection. Our CS selection methods may favour those sequences, since they may be more similar to our participant consensus because of convergent evolution. Using such a CS may infer a root that is at the middle of infection.

Our findings have implications beyond pure ancestral sequence reconstruction itself. If founder viruses could be reliably inferred from proviral sequences collected during chronic infection, then prevention relevant analyses that currently depend on rare acute-infection cohorts could potentially be extended to much larger and more routinely sampled populations. Our results suggest that this is not yet possible with sufficient reliability using current phylogenetic approaches, particularly when the sampled proviral reservoir lacks sequences close to the founder lineage. This helps define an important practical boundary for the use of chronic proviral data in studies of transmission bottlenecks, resistance transmission, vaccine relevant founder variants, and population-level HIV genomic surveillance.

In this study, we phylogenetically inferred founder virus sequences solely from HIV proviral sequences collected in chronic infection, with variable success. In particular, we were able to attain higher accuracy when our data set included a sequence that was similar to the founder sequence. Overall, our findings highlight the need for new methods of ancestral sequence reconstruction for estimating HIV founder virus sequences from proviral diversity sampled during chronic infection. Ideally, these methods will incorporate recombination and indels and possibly make use of hypermutated sequences. Due to low bootstrap support values in phylogenetic reconstruction (e.g. the bootstraps in this study were low as shown in Figs S9 and S10), integrating through multiple phylogenies (such as through Bayesian sampling) give us a better grasp on our confidence than maximum likelihood and parsimony methods. However, in our study, using MrBayes (Ronquist et al. 2012) to simultaneously infer topology and ancestral sequence did not provide any improvement over methods on a single tree topology. Using other techniques not explored here, for example generative AI (De Leonardis et al. 2025) or a full Bayesian inference (Baele et al. 2025), could provide more accurate results. In short, more sophisticated methods will need to be developed to successfully address the problem of founder sequence reconstruction from proviral sequences alone.

## Supplementary Material

Supplementary_materials_veag025

## Data Availability

The sequences in this study were previously published on Genbank and the Los Alamos National Laboratory HIV Sequence Database. Genbank accession numbers for all sequences used are provided in Table S13. Custom scripts used in this study are available on Zenodo (https://doi.org/10.5281/zenodo.19615897).
