# Supplementary material for: Getting to the root of HIV transmitted founder virus sequences
Source: Virus Evol. 2026 Apr 20;12(1):veag025. doi: 10.1093/ve/veag025 (PMC13155111; doi:10.1093/ve/veag025)
Supplement: Supplementary_materials_veag025 [file supplementary_materials_veag025.pdf]

Supplementary Materials: Getting to the root of HIV transmitted founder virus sequences

Bradley R. Jones, Zabrina L. Brumme, Eric Hunter, Jeffrey B. Joy

Supplementary Figures

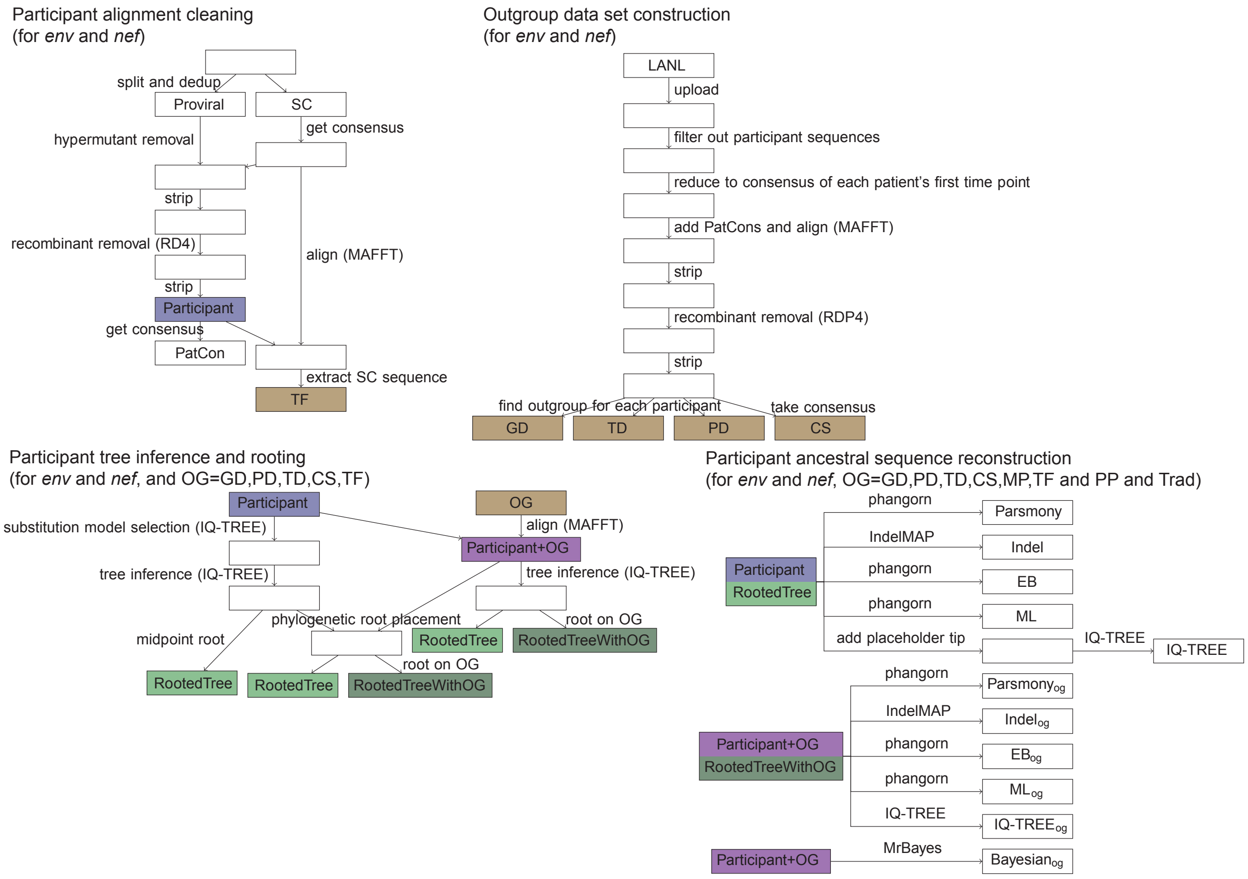

Supplementary Figure 1. Project pipelines. Edges are labelled by action performed and software used and important nodes are labelled. Participant pipelines were performed on each participant separately. PP: phylogenetic placement, Trad: traditional outgroup rooting.

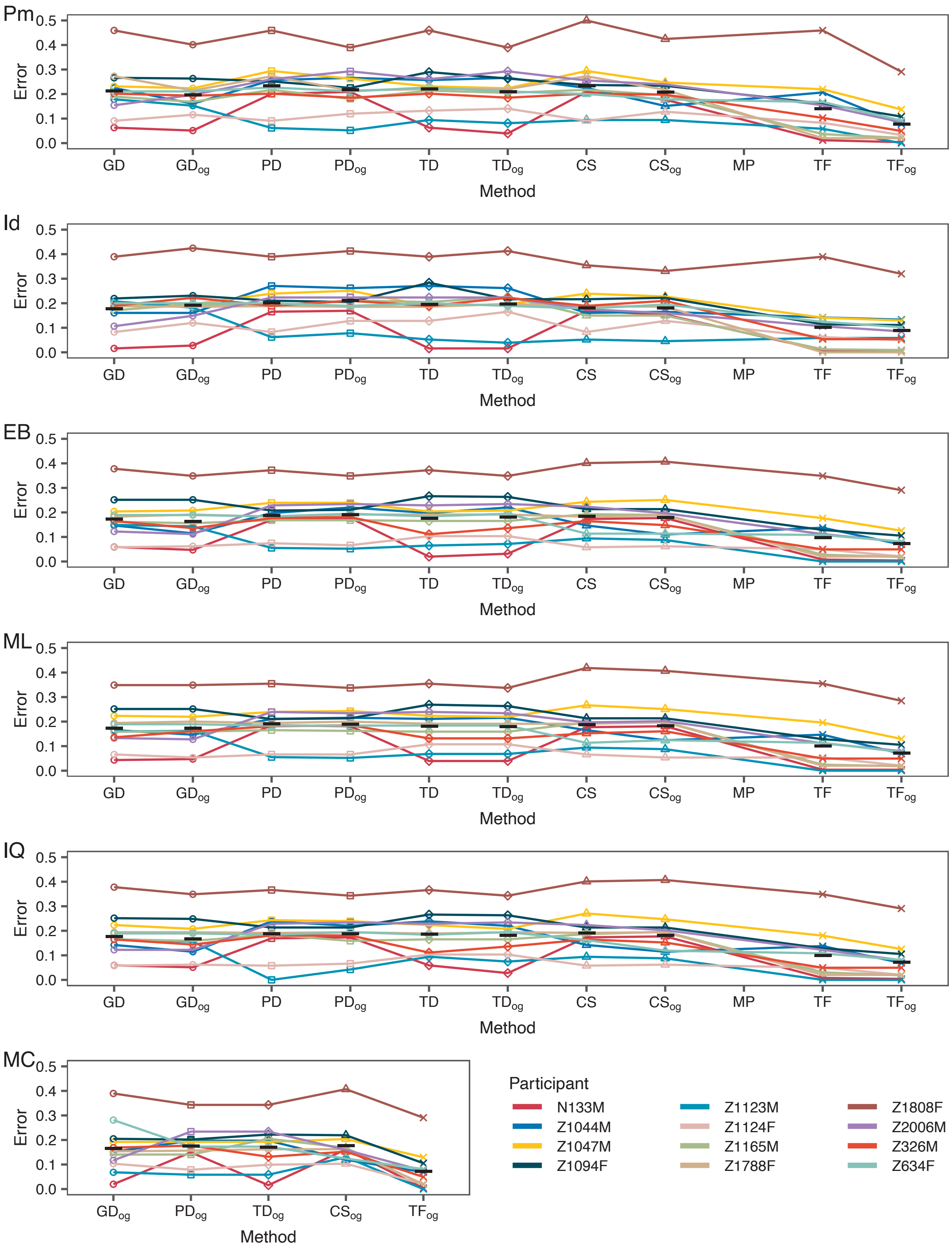

Pm
Id
EB
ML
IQ
MC
Error
Method
GD
GD$_{og}$
PD
PD$_{og}$
TD
TD$_{og}$
CS
CS$_{og}$
MP
TF
TF$_{og}$
0.0
0.1
0.2
0.3
0.4
0.5
Participant
N133M
Z1044M
Z1047M
Z1094F
Z1123M
Z1124F
Z1165M
Z1788F
Z1808F
Z2006M
Z326M
Z634F

Supplementary Figure 2. Error of ancestral sequence reconstruction methods of *env* (no phylogenetic placement). Error is number of incorrect bases divided by the number of variable sites (see description in the Materials and Methods). Colour corresponds to participant and lines link replicates from the same participant. Black horizontal bars indicate the mean error for each outgroup. Pm: parsimony with phangorn; ID: parsimony with indelMAP, EB: empirical Bayes with phangorn, ML: maximum likelihood with phangorn, IQ: maximum likelihood with IQ-TREE, MC: Markov chain Monte Carlo with MrBayes. GD: genetic distance root, PD: phylogenetic distance root, TD: topological distance root, CS: consensus sequence root, MP: midpoint rooting, TF: founder sequence root; og: ancestral reconstructions that included the outgroup sequence.

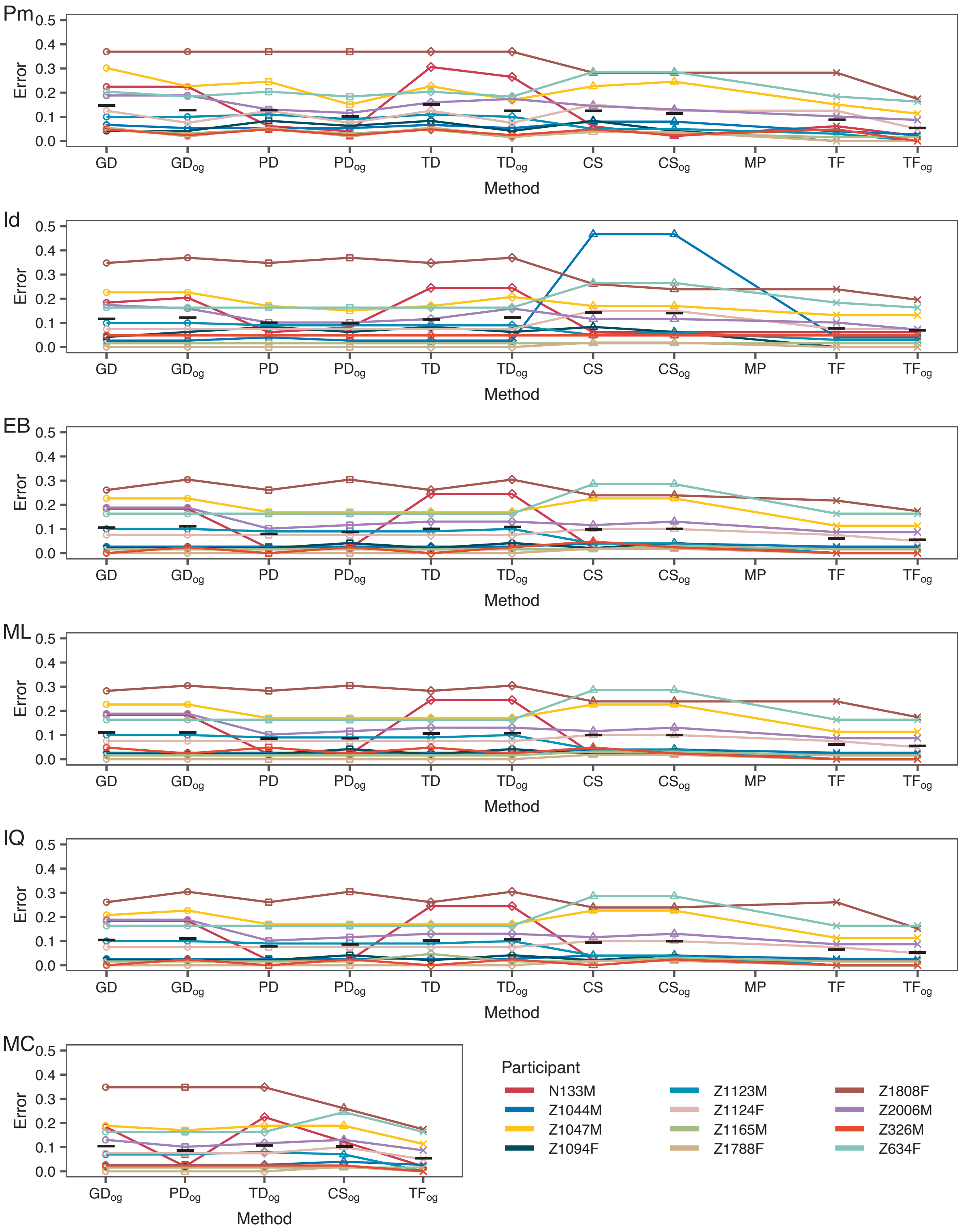

Pm
Id
EB
ML
IQ
MC
Error
0.0
0.1
0.2
0.3
0.4
0.5
GD
GDog
PD
PDog
TD
TDog
CS
CSog
MP
TF
TFog
Method
Participant
N133M
Z1044M
Z1047M
Z1094F
Z1123M
Z1124F
Z1165M
Z1788F
Z1808F
Z2006M
Z326M
Z634F

Supplementary Figure 3. Error of ancestral sequence reconstruction methods of *nef* (no phylogenetic placement). Error is number of incorrect bases divided by the number of variable sites (see description in the Materials and Methods). Colour corresponds to participant and lines link replicates from the same participant. Black horizontal bars indicate the mean error for each outgroup. Pm: parsimony with phangorn; ID: parsimony with indelMAP, EB: empirical Bayes with phangorn, ML: maximum likelihood with phangorn, IQ: maximum likelihood with IQ-TREE, MC: Markov chain Monte Carlo with MrBayes. GD: genetic distance root, PD: phylogenetic distance root, TD: topological distance root, CS: consensus sequence root, MP: midpoint rooting, TF: founder sequence root; og: ancestral reconstructions that included the outgroup sequence.

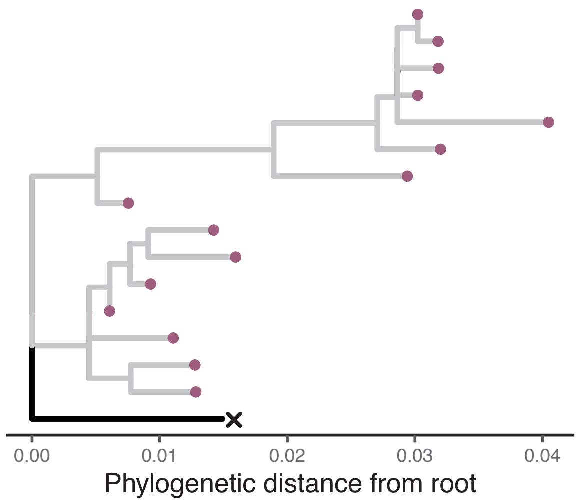

Supplementary Figure 4. *Nef* phylogenetic tree of participant Z1808F with proxy founder sequence inserted with phylogenetic placement. Founder sequence terminal branch is coloured black. Phylogenetic distance is in substitutions per site. Note how the length of the terminal branch of the founder sequence is more than a third of the root to tip distance of the most divergent sequence from the root (the sequence on the far right).

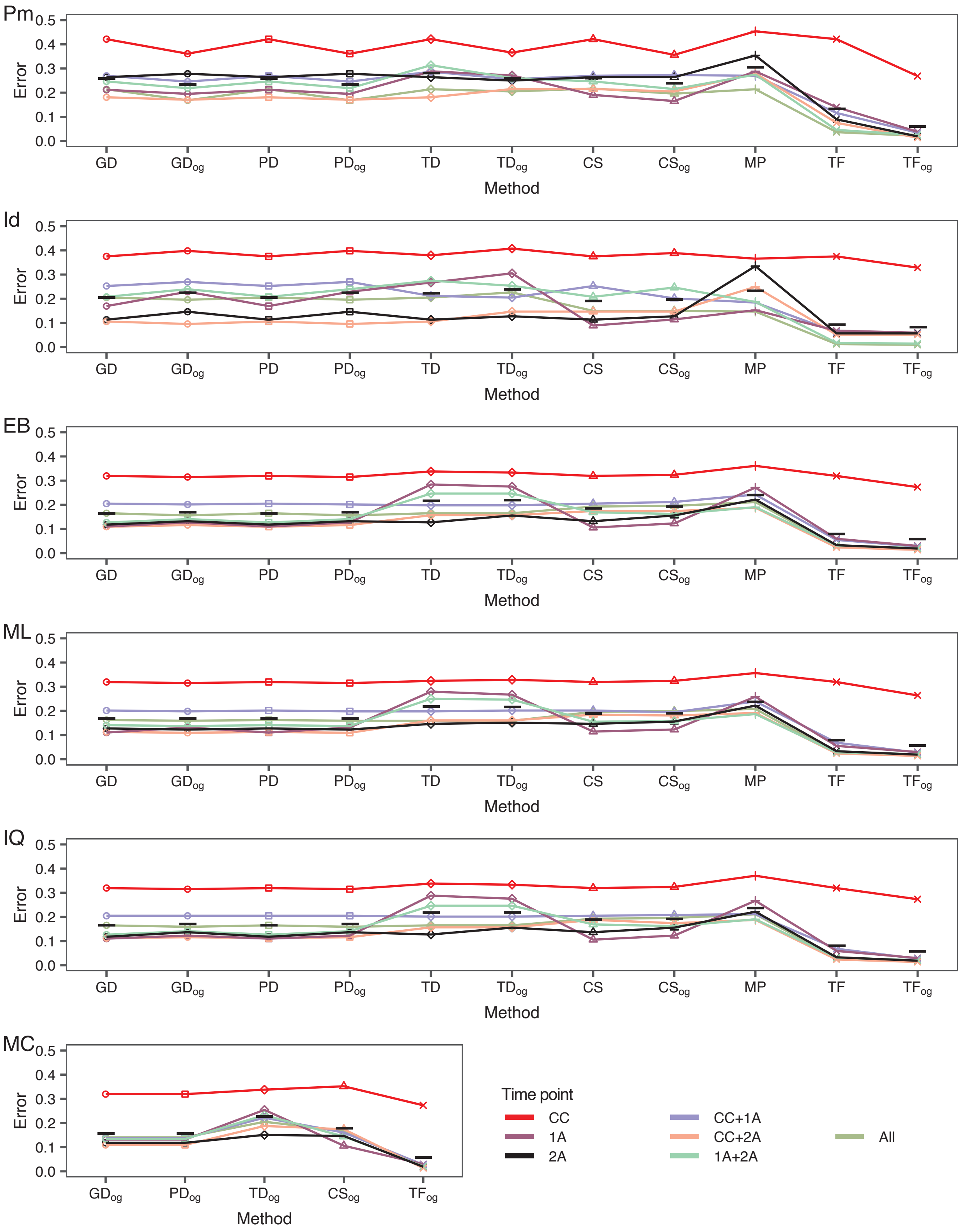
Pm
Id
EB
ML
IQ
MC
Error
0.0 0.1 0.2 0.3 0.4 0.5
GD GD_{og} PD PD_{og} TD TD_{og} CS CS_{og} MP TF TF_{og}
Method
GD_{og} PD_{og} TD_{og} CS_{og} TF_{og}
Time point
CC
1A
2A
CC+1A
CC+2A
1A+2A
All

Supplementary Figure 5. Error of ancestral sequence reconstruction methods of Z1165M with time points removed (*env*). Error is number of incorrect bases divided by the number of variable sites (see description in the Materials and Methods). Colour corresponds to retained time points and lines link replicates from the same data set. Black horizontal bars indicate the mean error for each outgroup. Pm: parsimony with phangorn; ID: parsimony with indelMAP, EB: empirical Bayes with phangorn, ML: maximum likelihood with phangorn, IQ: maximum likelihood with IQ-TREE, MC: Markov chain Monte Carlo with MrBayes. GD: genetic distance root, PD: phylogenetic distance root, TD: topological distance root, CS: consensus sequence root, MP: midpoint rooting, TF: founder sequence root; og: ancestral reconstructions that included the outgroup sequence.

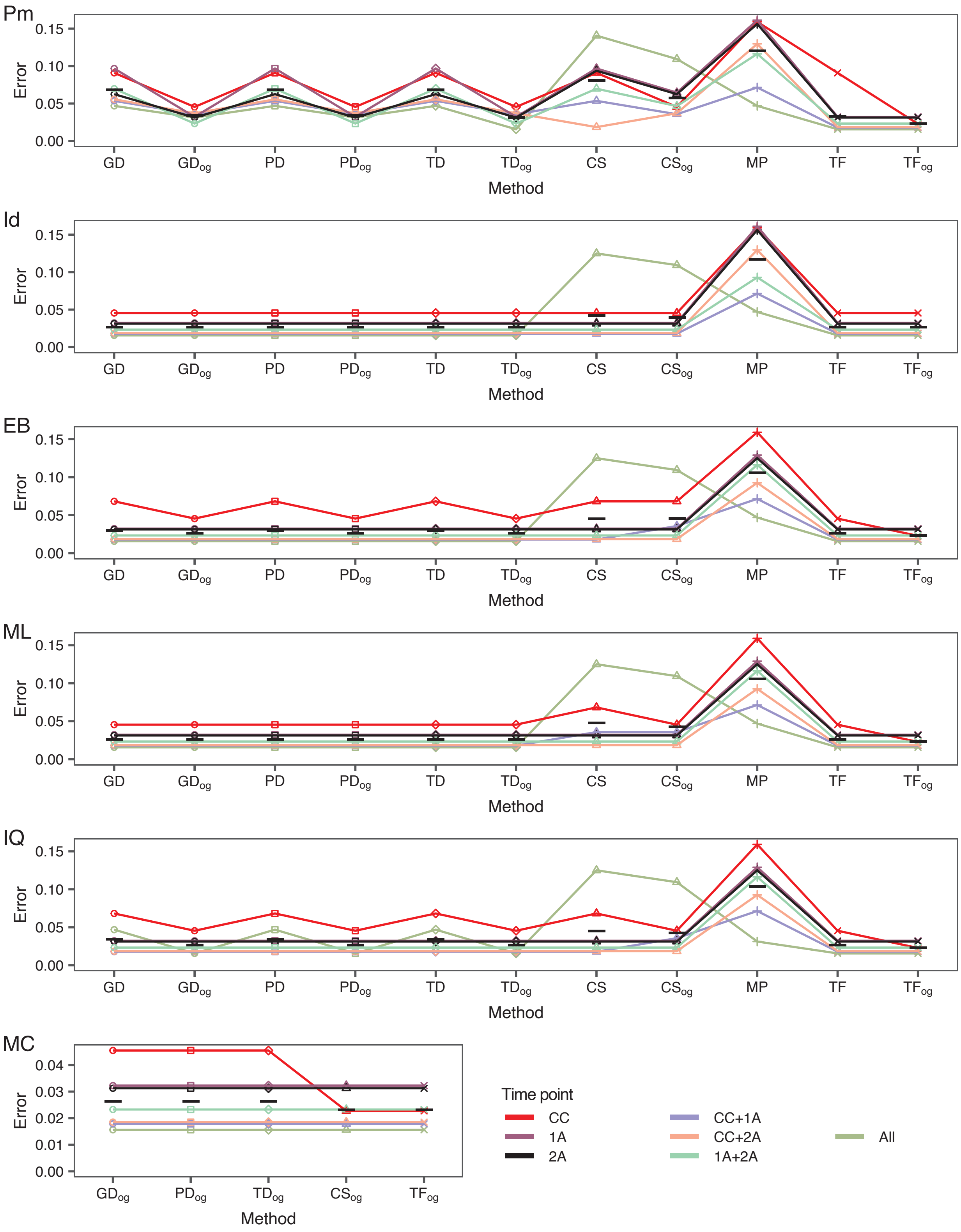
Pm
Id
EB
ML
IQ
MC
Error
0.00
0.05
0.10
0.15
0.01
0.02
0.03
0.04
GD
GDog
PD
PDog
TD
TDog
CS
CSog
MP
TF
TFog
Method
Time point
CC
1A
2A
CC+1A
CC+2A
1A+2A
All

Supplementary Figure 6. Error of ancestral sequence reconstruction methods of Z1165M with time points removed (*nef*). Error is number of incorrect bases divided by the number of variable sites (see description in the Materials and Methods). Colour corresponds to retained time points and lines link replicates from the same data set. Black horizontal bars indicate the mean error for each outgroup. Pm: parsimony with phangorn; ID: parsimony with indelMAP, EB: empirical Bayes with phangorn, ML: maximum likelihood with phangorn, IQ: maximum likelihood with IQ-TREE, MC: Markov chain Monte Carlo with MrBayes. GD: genetic distance root, PD: phylogenetic distance root, TD: topological distance root, CS: consensus sequence root, MP: midpoint rooting, TF: founder sequence root; og: ancestral reconstructions that included the outgroup sequence.

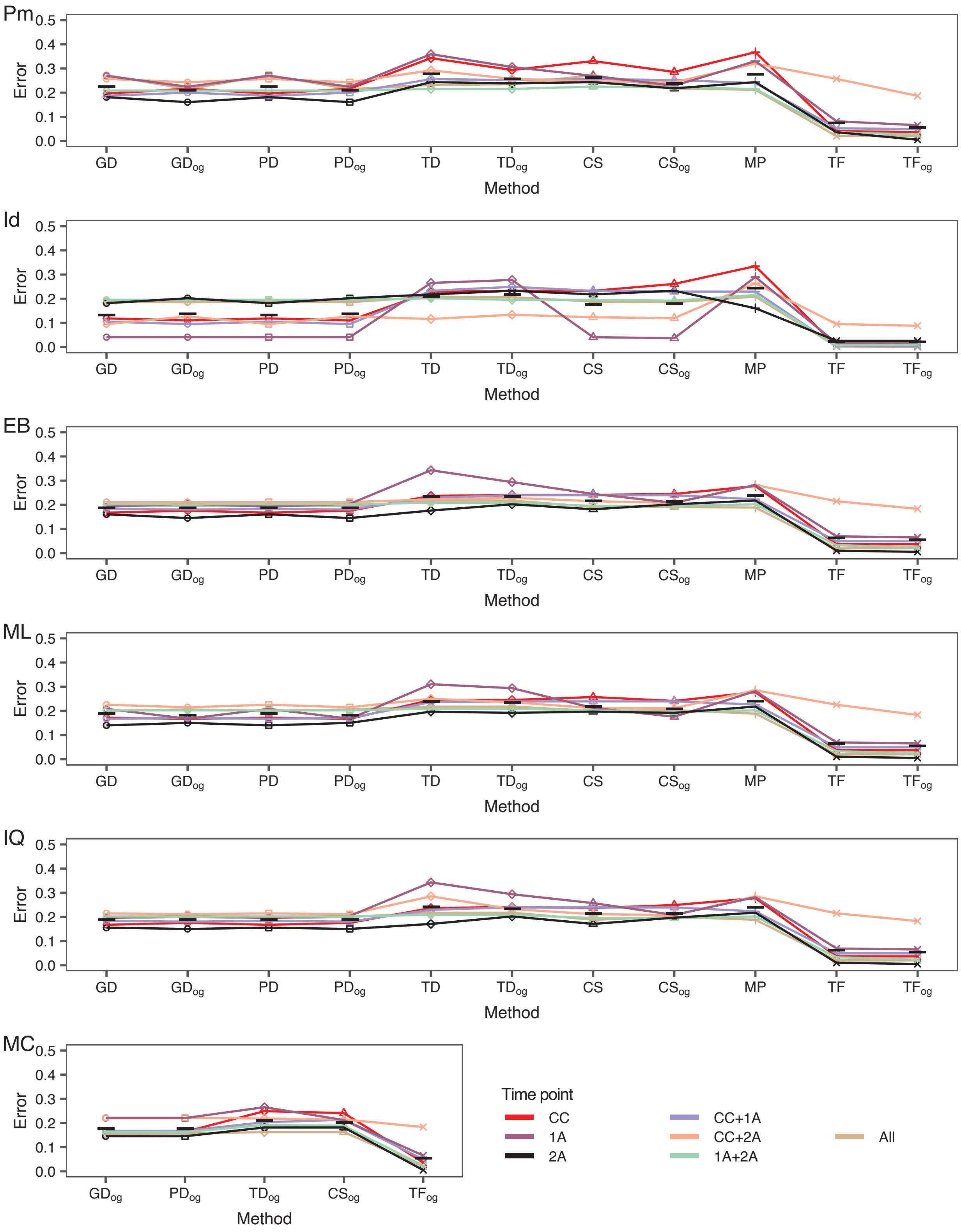
Pm
Id
EB
ML
IQ
MC
Error
0.0
0.1
0.2
0.3
0.4
0.5
GD
GDog
PD
PDog
TD
TDog
CS
CSog
MP
TF
TFog
Method
Time point
CC
1A
2A
CC+1A
CC+2A
1A+2A
All

Supplementary Figure 7. Error of ancestral sequence reconstruction methods of Z1788F with time points removed (*env*). Error is number of incorrect bases divided by the number of variable sites (see description in the Materials and Methods). Colour corresponds to retained time points and lines link replicates from the same data set. Black horizontal bars indicate the mean error for each outgroup. Pm: parsimony with phangorn; ID: parsimony with indelMAP, EB: empirical Bayes with phangorn, ML: maximum likelihood with phangorn, IQ: maximum likelihood with IQ-TREE, MC: Markov chain Monte Carlo with MrBayes. GD: genetic distance root, PD: phylogenetic distance root, TD: topological distance root, CS: consensus sequence root, MP: midpoint rooting, TF: founder sequence root; og: ancestral reconstructions that included the outgroup sequence.

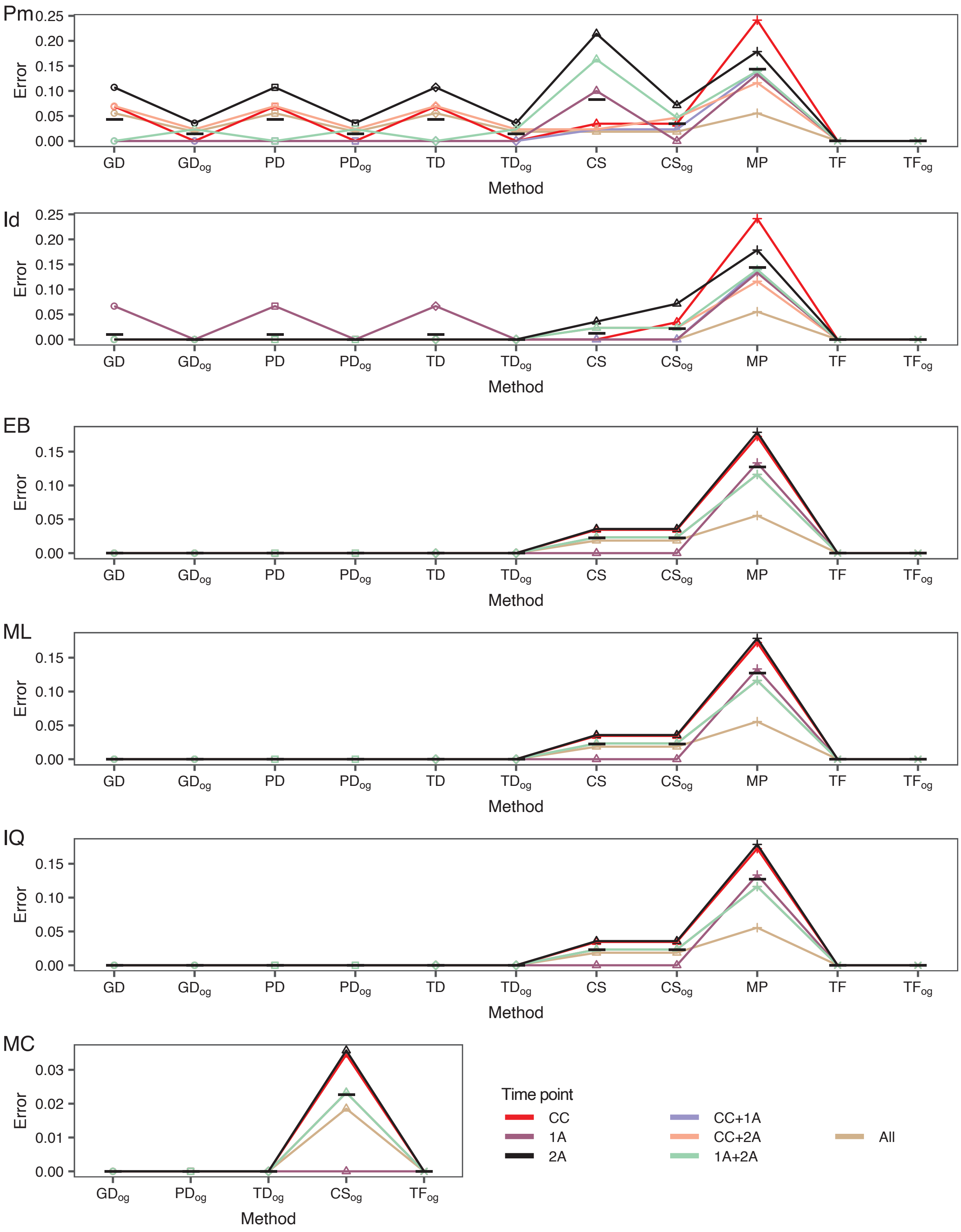
Pm
Id
EB
ML
IQ
MC
Error
0.00
0.05
0.10
0.15
0.20
0.25
0.01
0.02
0.03
GD
GD$_{og}$
PD
PD$_{og}$
TD
TD$_{og}$
CS
CS$_{og}$
MP
TF
TF$_{og}$
Method
Time point
CC
1A
2A
CC+1A
CC+2A
1A+2A
All

Supplementary Figure 8. Error of ancestral sequence reconstruction methods of Z1788F with time points removed (*nef*). Error is number of incorrect bases divided by the number of variable sites (see description in the Materials and Methods). Colour corresponds to retained time points and lines link replicates from the same data set. Black horizontal bars indicate the mean error for each outgroup. Pm: parsimony with phangorn; ID: parsimony with indelMAP, EB: empirical Bayes with phangorn, ML: maximum likelihood with phangorn, IQ: maximum likelihood with IQ-TREE, MC: Markov chain Monte Carlo with MrBayes. GD: genetic distance root, PD: phylogenetic distance root, TD: topological distance root, CS: consensus sequence root, MP: midpoint rooting, TF: founder sequence root; og: ancestral reconstructions that included the outgroup sequence.

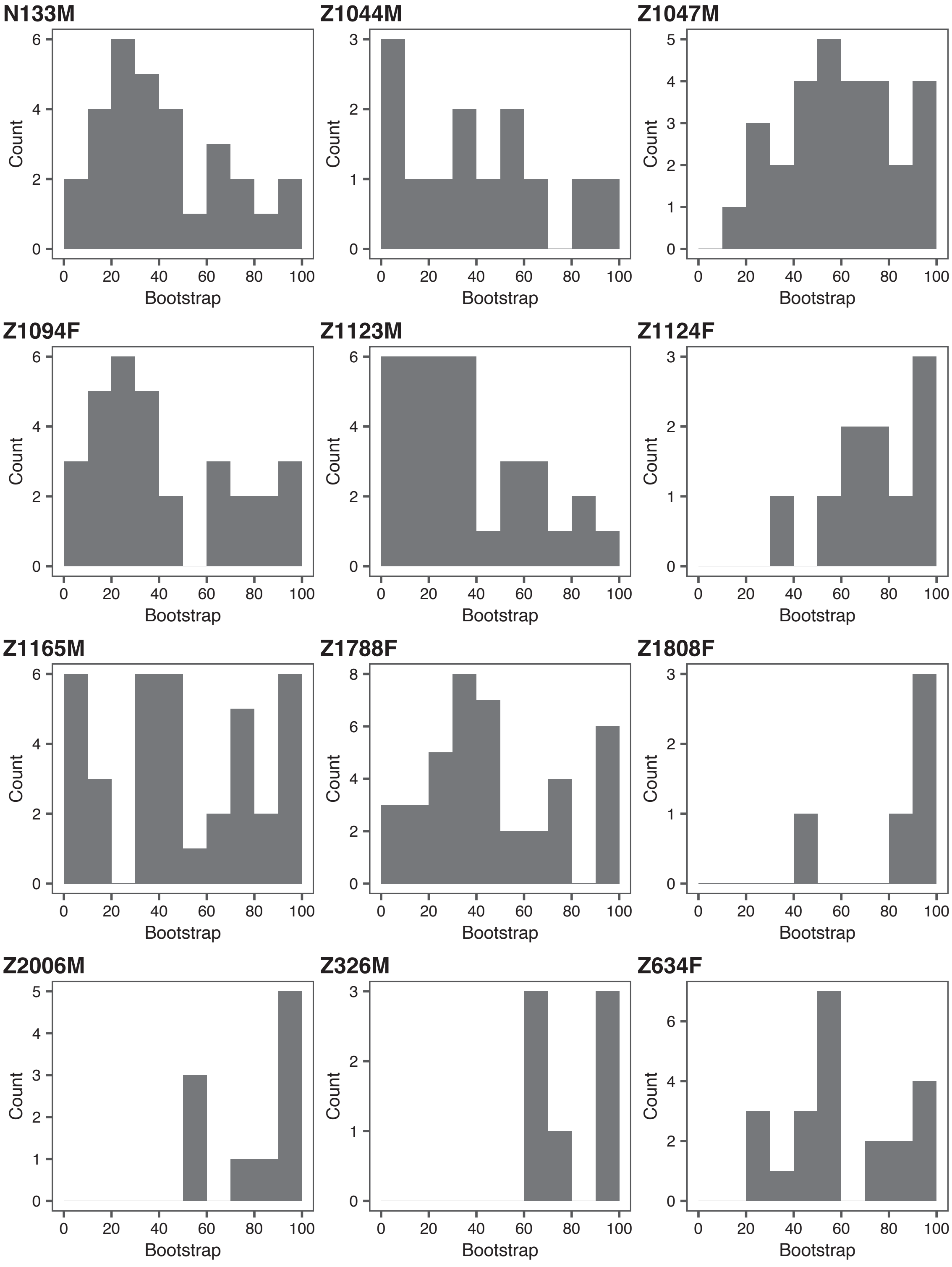

Supplementary Figure 9. Distribution of phylogenetic bootstrap support values for each split for each participant phylogeny (*env*).

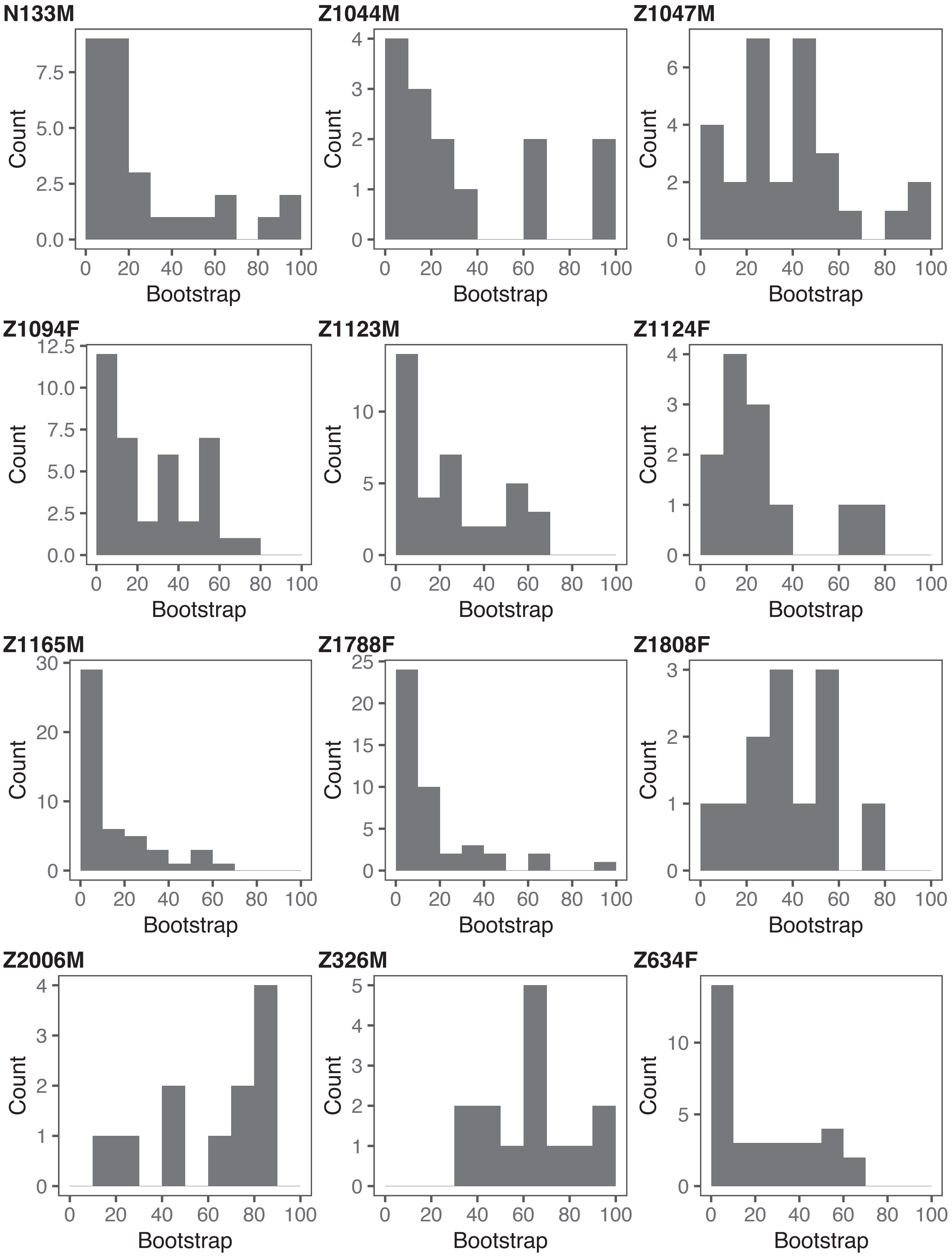

Supplementary Figure 10. Distribution of phylogenetic bootstrap support values for each split for each participant phylogeny (*nef*).

Supplementary Tables

Supplementary Table 1. Best fitting substitution models for *env*. Best fitting substitution models for *env* selected by IQ-TREE for participant / ancestral sequence reconstruction software.

Participant	IQ-TREE	Phangorn	IndelMap	MrBayes
N133M	K3PU+F+R3	TIM+F+I+G4	TIM+F	GTR+F+I+G4
Z1044M	K3Pu+F+I+G4	K3Pu+F+I+G4	K3Pu+F	GTR+F+I+G4
Z1047M	K3Pu+F+R3	K3Pu+F+I+G4	K3Pu+F	HKY+F+I+G4
Z1094F	TIM+F+R4	GTR+F+I+G4	K3Pu +F	GTR+F+I+G4
Z1123M	TIM+F+R3	GTR+F+I+G4	GTR+F	GTR+F+I+G4
Z1124F	TVM+F+I+G4	TVM+F+I+G4	TVM+F	GTR+F+I+G4
Z1165M	GTR+F+I+G4	GTR+F+I+G4	GTR+F	GTR+F+I+G4
Z1788F	TVM+F+R3	GTR+F+I+G4	TVM+F	GTR+F+I+G4
Z1808F	HKY+F+I	HKY+F+I	HKY+F	HKY+F+I
Z2006M	K3Pu+F+I	K3Pu+F+I	K3Pu+F	GTR+F+I
Z326M	K3Pu+F+I	K3Pu+F+I	K3Pu+F	GTR+F+I
Z634F	GTR+F+I+G4	GTR+F+I+G4	GTR+F	GTR+F+I+G4

Supplementary Table 2. Best fitting substitution models for *nef*. Best fitting substitution models for *env* selected by IQ-TREE for participant / ancestral sequence reconstruction software.

Participant	IQ-TREE	Phangorn	IndelMap	MrBayes
N133M	HKY+F+I+G4	HKY+F+I+G4	HKY+F	HKY+F+I+G4
Z1044M	HKY+F+I	HKY+F+I	HKY+F	HKY+F+I
Z1047M	HKY+F+I+G	HKY+F+I+G4	TN+F	HKY+F+I+G4
Z1094F	HKY+F+I	HKY+F+I	HKY+F	HKY+F+I
Z1123M	TPM3u+F+I	TPM3u+F+I+	TPM3u+F	HKY+F+I
Z1124F	HKY+F+I	HKY+F+I	HKY+F	HKY+F+I

Z1165M	TPM2u+F+I+G4	TPM2u+F+I+G4	TPM2u+F	HKY+F+I+G4
Z1788F	HKY+F+R3	HKY+F+I+G4	TPM2u+F	HKY+F+I+G4
Z1808F	K3Pu+F+I	K3PU+F+I	K3Pu+F	HKY+F+I
Z2006M	HKY+F+I	HKY+F+I	HKY+F	HKY+F+I
Z326M	TPM2u+F+I	TPM2u+F+I	TPM2u+F	HKY+F+I
Z634F	TPM2u+F+R3	TPM2u+F+I+G4	TPM2u+F	HKY+F+I+G4

Supplementary Table 3. Number of distinct sequences (*env*). SC: plasma sample used to make proxy fonder sequence. CC: proviral sample shortly before cART initiation. 1A: first proviral sample after cART initiation. 2A second proviral sample after cART initiation. Hypermutants: hypermutant sequences detected. Recombinants: recombinant sequences detected. Cleaned Proviral: number of proviral sequences after removing hypermutants and recombinants. Note: some distinct sequences appeared in multiple time points.

Participant	SC	CC	1A	2A	Hypermutants	Recombinants	Cleaned Proviral
N133M	20	0	19	16	0	1	33
Z1044M	8	0	17	0	0	1	16
Z1047M	5	21	20	0	0	9	32
Z1094F	13	23	21	0	0	10	34
Z1123M	4	19	23	0	0	4	38
Z1124F	5	0	15	0	0	2	13
Z1165M	11	23	17	14	0	14	40
Z1788F	8	19	20	16	0	11	43
Z1808F	8	0	16	0	1	7	8
Z2006M	4	0	14	0	1	0	13
Z326M	9	0	17	0	0	7	10
Z634F	7	0	22	14	7	4	25
Total	102	105	222	60	9	70	305

Supplementary Table 4. Number of distinct sequences (*nef*). SC: plasma sample used to make proxy fonder sequence. CC: proviral sample shortly before cART initiation. 1A: first proviral sample after cART initiation. 2A second proviral sample after cART initiation. Hypermutants: hypermutant sequences detected. Recombinants: recombinant sequences detected. Cleaned Proviral: number of proviral sequences after removing hypermutants and recombinants. Note: some distinct sequences appeared in multiple time points.

Participant	SC	CC	1A	2A	Hypermutants	Recombinants	Cleaned Proviral
N133M	7	0	17	16	0	0	32
Z1044M	6	0	17	0	0	0	17
Z1047M	2	15	19	0	0	0	32
Z1094F	3	21	20	0	0	0	41
Z1123M	1	18	23	0	0	0	40
Z1124F	4	0	15	0	0	0	15
Z1165M	4	23	16	14	0	0	51
Z1788F	4	18	19	15	0	0	47
Z1808F	1	0	15	0	0	0	15
Z2006M	6	0	14	0	0	0	14
Z326M	4	0	17	0	0	0	17
Z634F	7	0	21	14	0	0	35
Total	49	95	213	59	0	0	356

Supplementary Table 5. Root placement distance from founder root in *env*. Topo: topological distance (number of branches between root and founder root), evol: evolutionary distance (phylogenetic distance between root and founder root in substitutions per site). MP: midpoint root, CS: consensus sequence root, GD: genetic distance root, PD: phylogenetic distance root, TD: topological distance root.

Participant	MP	CS	GD	PD	TD

	Topo	Evol	Topo	Evol	Topo	Evol	Topo	Evol	Topo	Evol
N133M	11	1.73e-2	3	2.93e-3	2	3.61e-3	15	2.78e-2	2	3.26e-3
Z1044M	0	4.82e-3	0	2.16e-3	0	1.79e-3	8	2.14e-2	8	2.14e-2
Z1047M	4	1.54e-2	8	1.82e-2	3	1.00e-2	3	1.00e-2	3	1.00e-2
Z1094F	4	1.29e-2	6	1.82e-2	11	2.89e-2	7	2.01e-2	12	3.21e-2
Z1123M	9	2.27e-2	8	2.02e-2	1	1.01e-2	1	1.01e-2	1	1.01e-2
Z1124F	5	2.15e-2	1	3.38e-3	1	3.44e-3	1	3.65e-3	3	8.70e-3
Z1165M	6	3.41e-2	6	3.27e-2	6	2.78e-2	6	2.78e-2	6	2.72e-2
Z1788F	12	2.99e-2	13	3.31e-2	13	3.39e-2	13	3.39e-2	17	4.44e-2
Z1808F	1	8.14e-3	1	1.25e-2	0	1.91e-3	0	1.11e-3	0	1.11e-3
Z2006M	1	2.87e-3	2	1.11e-2	1	1.95e-3	4	1.45e-2	1	4.34e-3
Z326M	4	2.94e-2	2	1.04e-2	2	1.03e-2	2	1.17e-2	2	9.36e-3
Z634F	4	1.50e-2	1	2.59e-3	4	1.39e-2	2	9.42e-3	2	9.42e-3

Supplementary Table 6. Root placement distance from founder root in *nef*. Topo: topological distance (number of branches between root and founder root), evol: evolutionary distance (phylogenetic distance between root and founder root in substitutions per site). GD: genetic distance root, PD: phylogenetic distance root, TD: topological distance root.

**Participant**	**MP**		**CS**		**GD**		**PD**		**TD**	
	Topo	Evol	Topo	Evol	Topo	Evol	Topo	Evol	Topo	Evol
N133M	4	8.83e-3	11	2.80e-2	10	2.13e-2	0	8.51e-4	12	2.81e-2
Z1044M	3	6.50e-3	1	1.64e-3	1	4.95e-5	1	4.95e-5	1	5.01e-5
Z1047M	3	9.04e-3	3	1.29e-2	8	2.91e-2	2	8.02e-3	3	7.57e-3
Z1094F	4	3.86e-3	4	4.49e-3	3	1.65e-3	4	4.44e-3	4	4.48e-3
Z1123M	2	6.86e-3	2	6.83e-3	8	1.50e-2	8	1.86e-2	2	6.83e-3
Z1124F	3	7.38e-3	2	3.25e-3	0	1.58e-3	0	1.58e-3	0	1.58e-3
Z1165M	6	3.16e-3	15	1.37e-2	1	8.82e-6	1	8.82e-6	3	1.14e-5
Z1788F	5	4.74e-3	2	1.62e-3	0	7.01e-6	0	7.01e-6	0	7.01e-6
Z1808F	1	1.16e-2	1	6.99e-3	0	1.76e-3	0	1.76e-3	0	1.76e-3
Z2006M	2	4.74e-3	5	1.10e-2	5	1.06e-2	4	9.53e-3	3	8.30e-3
Z326M	4	1.53e-2	0	3.22e-3	0	3.18e-3	0	3.18e-3	0	3.18e-3
Z634F	6	1.65e-2	6	1.56e-2	3	8.64e-3	3	8.64e-3	3	8.64e-3

Supplementary Table 7. Root mean square deviation of root-to-tip distances between rooted trees with unfixed topology in *env*. CS: consensus sequence root, GD: genetic distance root,

PD: phylogenetic distance root, TD: topological distance root, Fixed TF: fixed phylogeny with founder virus root. (Units in substitutions per site).

Participant	CS	GD	PD	TD	Fixed TF
N133M	9.98e-3	1.36e-3	9.59e-3	1.12e-3	1.63e-5
Z1044M	3.16e-3	3.04e-3	1.00e-2	1.00e-2	1.87e-3
Z1047M	1.19e-2	1.27e-2	1.27e-2	1.27e-2	3.21e-4
Z1094F	1.44e-2	9.44e-3	1.38e-2	8.58e-3	4.95e-3
Z1123M	4.68e-3	1.33e-2	6.35e-3	5.65e-3	5.10e-4
Z1124F	3.79e-3	4.32e-3	4.03e-3	9.58e-3	5.51e-4
Z1165M	1.72e-2	2.18e-2	2.50e-2	1.95e-2	9.81e-4
Z1788F	2.36e-2	2.31e-2	2.31e-2	2.08e-2	1.82e-5
Z1808F	1.05e-2	3.82e-3	3.31e-3	3.33e-3	1.02e-3
Z2006M	1.14e-2	1.41e-3	1.33e-2	1.33e-2	1.35e-3
Z326M	1.02e-2	1.02e-2	1.20e-2	9.74e-3	4.01e-6
Z634F	2.51e-3	7.77e-3	1.00e-2	1.00e-2	7.02e-4

Supplementary Table 8. Root mean square deviation of root-to-tip distances between rooted trees with unfixed topology in *nef*. GD: genetic distance root, PD: phylogenetic distance root, TD: topological distance root, Fixed TF: fixed phylogeny with founder virus root. (Units in substitutions per site).

Participant	CS	GD	PD	TD	Fixed TF
N133M	2.84e-3	9.66e-3	5.12e-3	1.13e-2	1.02e-3
Z1044M	5.91e-3	5.22e-5	6.67e-3	5.32e-5	8.66e-5
Z1047M	1.21e-2	1.35e-2	1.08e-2	1.03e-2	3.34e-3
Z1094F	4.04e-3	3.09e-3	3.52e-3	4.13e-3	1.56e-3
Z1123M	8.14e-3	9.25e-3	8.32e-3	7.81e-3	3.83e-3
Z1124F	3.23e-3	1.61e-3	1.61e-3	1.61e-3	1.35e-4
Z1165M	7.22e-3	6.31e-3	6.48e-3	6.80e-3	6.16e-3
Z1788F	6.43e-3	5.71e-3	4.83e-3	5.78e-3	5.59e-3
Z1808F	7.53e-3	1.23e-2	1.23e-2	1.23e-2	9.29e-4
Z2006M	8.80e-3	1.08e-2	7.37e-3	5.89e-3	2.65e-4
Z326M	3.83e-3	3.47e-3	3.47e-3	3.47e-3	1.20e-2
Z634F	6.96e-3	1.01e-2	9.85e-3	1.01e-2	1.20e-3

Supplementary Table 9. Transmitted founder sequence terminal branch length in e*nv*. Units in substitutions per site.

Participant	Fixed tree	Unfixed tree
N133M	1.03e-6	1.00e-6
Z1044M	7.28e-3	7.27e-3
Z1047M	5.27e-3	5.28e-3
Z1094F	1.46e-3	1.45e-3
Z1123M	1.02e-6	1.01e-6
Z1124F	2.41e-3	2.41e-3
Z1165M	1.07e-6	1.92e-6
Z1788F	1.04e-6	1.00e-6
Z1808F	1.40e-2	1.40e-2
Z2006M	4.53e-3	4.53e-3
Z326M	1.00e-6	1.00e-6
Z634F	4.10e-3	4.10e-3

Supplementary Table 10. Transmitted founder sequence terminal branch length in *nef*. Units in substitutions per site.

Participant	Fixed tree	Unfixed tree
N133M	4.83e-7	5.07e-7
Z1044M	3.29e-3	3.29e-3
Z1047M	9.46e-3	9.45e-3
Z1094F	1.00e-6	1.00e-6
Z1123M	1.09e-6	1.00e-6
Z1124F	3.35e-3	3.35e-3
Z1165M	7.07e-7	9.31e-7
Z1788F	1.01e-6	1.02e-6
Z1808F	1.49e-2	1.49e-2
Z2006M	8.56e-3	8.56e-3
Z326M	1.00e-6	1.00e-6
Z634F	1.52e-2	1.52e-2

Supplementary Table 11. Missing columns (indels) in TF sequence for *env*. TF missing: number of missing columns in the TF sequence. Variable sites: number of columns in the alignment of TF and proviral sequences that have more than one type of base and / or missing base in at

least one sequence (this is the denominator of the ancestral sequence error). Total length: the number of columns in the alignment of TF and proviral sequences.

Participant	TF missing	Variable sites	Total length
N133M	1	254	2559
Z1044M	11	218	2520
Z1047M	27	255	2523
Z1094F	34	342	2583
Z1123M	0	308	2601
Z1124F	6	242	2547
Z1165M	6	327	2562
Z1788F	7	345	2575
Z1808F	34	172	2573
Z2006M	9	188	2566
Z326M	12	243	2589
Z634F	9	185	2514

Supplementary Table 12. Missing columns (indels) in TF sequence for *nef*. TF missing: number of missing columns in the TF sequence. Variable sites: number of columns in the alignment of TF and proviral sequences that have more than one type of base and / or missing base in at least one sequence (this is the denominator of the ancestral sequence error). Total length: the number of columns in the alignment of TF and proviral sequences.

Participant	TF missing	Variable sites	Total length
N133M	1	49	621
Z1044M	2	75	621
Z1047M	4	53	624
Z1094F	0	48	624
Z1123M	0	100	624
Z1124F	2	40	624
Z1165M	1	64	639
Z1788F	0	54	630
Z1808F	4	46	624
Z2006M	1	69	630
Z326M	0	83	660
Z634F	5	49	624

Supplementary Table 13. Sequence availability.

Dataset	Accession numbers
Zambia-Emory HIV Research Project	MT194125-MT194145, MT194167-MT194177, MT194222-MT194245, MT194282-MT194288, MT194290-MT194326, MT194328-MT194376, MT194458-MT194495, MT194497-MT194524, MT194581-MT194609, MT194674-MT194713, MT194738-MT194748, MT194750-MT194771, MT194898-MT194921, MT194957-MT194991, MT194993-MT195011, MT195034-MT195060, MT195134-MT195143, MT195145-MT195155, MT195184-MT195204, MT195232-MT195240, MT195242-MT195258, MT195316-MT195329, MT195364-MT195402, MT195425-MT195444, MT195493-MT195514, MT195516-MT195535
Reference (*env*)	AB254141-AB254156, AB485645-AB485647, AF286224-AF286225, AY423971, AY423984, AY424079, AY424138, AY424163, AY494971, AY805330, DQ388514-DQ388517, DQ422948, EU166353-EU166402, EU166413-EU166473, EU166483-EU166517, EU166548-EU166550, EU166554, EU166557-EU166558, EU166562-EU166566, EU166568-EU166569, EU166571-EU166572, EU166574, EU166576-EU166604, EU166653-EU166672, EU166674-EU166787, EU166789-EU166916, FJ496185-FJ496205, FJ496207, FJ496209-FJ496210, FJ496212-FJ496214, GQ485312-GQ485447, GU329048-GU329120, GU329131-GU329174, GU329184-GU329282, GU329284-GU329342, GU329354-GU329523, GU939049-GU939171, HM036739-HM036761, HM036763, HM036766-HM036767, HM036769-HM036771, HM036773-HM036775, HM036778-HM036780, HM036782-HM036819, HM036821-HM036830, HM036832, HM036835-HM036878, HM036880-HM036932, HM036983-HM037030, HM037032-HM037037, HM068598-HM068599, JN977604, JX213352-JX213473, JX239263-JX239282, JX239284-JX239290, JX239293-JX239294, JX239296, JX239298, JX239301, JX239303-JX239307, JX239311, JX239313-JX239323, JX239325, JX239329, JX239333, JX239337-JX239348, JX239350-JX239387, JX239389-JX239393, JX239395-JX239408, JX239410-JX239437, KF716466-KF716467, KP109494-KP109496, KR820294-KR820300, KR820302-KR820367, KR820369-KR820449, KT252545, KU200869-KU200897, KU200899-KU200911, KU200913-KU200943, KU749425-KU749426, KX983732-KX983929, KY229251-KY229377, KY229379-KY229383, KY229391-KY229467, KY229469-KY229518, KY229542-KY229595, KY229597-KY229682, MK749242-MK749296, MT194772, MT194774-MT194779, MT194781-MT194785, MT194788-MT194790, MT194792-MT194793, MT194796-MT194814, MT194816, MT194818-MT194897, MT347678-MT347681, ON890940-ON890941, ON890948-ON890949, ON890961, ON890964-ON890967, ON890972, ON890986, ON890991-ON890993, ON890997, ON891003, ON891011-ON891013, ON891024-ON891025, ON891043-ON891045, ON891049-ON891052, OQ747368-OQ747574
Reference (*nef*)	AB254141-AB254156, AB485645-AB485647, AF286224-AF286225, AY805330, DQ793058, DQ793060-DQ793068, DQ793070, FJ496185-FJ496205, FJ496207, FJ496209-FJ496210, FJ496212-FJ496214, KF716466-KF716467, KM049007, KM049009-KM049041, KM049050-KM049051, KM049053-KM049064,

	KM049071-KM049072, KM049074-KM049079, KM049087-KM049088, KM049098-KM049099, KM049107-KM049117, KM049125-KM049132, KM049134-KM049139, KM049141-KM049142, KM049158-KM049180, KM049182-KM049185, KM049187-KM049192, KM049194-KM049214, KM049216-KM049224, KM049226-KM049230, KM049232, KM049234-KM049243, KM049245, KM049248-KM049264, KM049266-KM049320, KM049322-KM049360, KM049362-KM049366, KM049368-KM049373, KM049381-KM049400, KM049402-KM049436, KM049438-KM049462, KM049464-KM049522, KM049529-KM049540, KM049542-KM049549, KM049551-KM049616, KM049618-KM049658, KM049661-KM049678, KM049680-KM049684, KM049687-KM049746, KM049753-KM049756, KM049758-KM049772, KM049774-KM049783, KM049785-KM049799, KM049801-KM049805, KM049807-KM049810, KM049812-KM049827, KM049829-KM049836, KM049838-KM049846, KM049849, KM049851-KM049854, KM049856-KM049857, KM049865-KM049867, KM049869-KM049880, KM049882-KM049899, KP109494-KP109496, KR820294-KR820300, KR820302-KR820367, KR820369-KR820449, KU749425-KU749426, MT194772, MT194774-MT194779, MT194781-MT194785, MT194788-MT194790, MT194792-MT194793, MT194796-MT194814, MT194816, MT194818-MT194897, MT347678-MT347681
LANL early transmission	AB254141-AB254156, AB485645-AB485647, AF286224-AF286225, AY423971, AY423984, AY424079, AY424138, AY424163, AY494971, AY805330, DQ388514-DQ388517, DQ422948, EU166353-EU166402, EU166413-EU166473, EU166483-EU166517, EU166548-EU166550, EU166554, EU166557-EU166558, EU166562-EU166566, EU166568-EU166569, EU166571-EU166572, EU166574, EU166576-EU166604, EU166653-EU166672, EU166674-EU166787, EU166789-EU166916, FJ496185-FJ496205, FJ496207, FJ496209-FJ496210, FJ496212-FJ496214, GQ485312-GQ485447, GU329048-GU329120, GU329131-GU329174, GU329184-GU329282, GU329284-GU329342, GU329354-GU329523, GU939049-GU939171, HM036739-HM036761, HM036763, HM036766-HM036767, HM036769-HM036771, HM036773-HM036775, HM036778-HM036780, HM036782-HM036819, HM036821-HM036830, HM036832, HM036835-HM036878, HM036880-HM036932, HM036983-HM037030, HM037032-HM037037, HM068598-HM068599, JN977604, JX213352-JX213473, JX239263-JX239282, JX239284-JX239290, JX239293-JX239294, JX239296, JX239298, JX239301, JX239303-JX239307, JX239311, JX239313-JX239323, JX239325, JX239329, JX239333, JX239337-JX239348, JX239350-JX239387, JX239389-JX239393, JX239395-JX239408, JX239410-JX239437, KF716466-KF716467, KP109494-KP109496, KR820294-KR820300, KR820302-KR820367, KR820369-KR820449, KT252545, KU200869-KU200897, KU200899-KU200911, KU200913-KU200943, KU749425-KU749426, KX983732-KX983929, KY229251-KY229377, KY229379-KY229383, KY229391-KY229467, KY229469-KY229518, KY229542-KY229595, KY229597-KY229682, MK749242-MK749296, MT194125-MT194132, MT194134-MT194140, MT194142-MT194225, MT194227-MT194230, MT194232-MT194240, MT194242-MT194295, MT194297-MT194298, MT194300-MT194312, MT194314-MT194340, MT194343-MT194366, MT194369-MT194461, MT194463-MT194470, MT194473, MT194475-

	MT194500, MT194502-MT194510, MT194512-MT194522, MT194524-MT194580, MT194582-MT194588, MT194590-MT194591, MT194594-MT194677, MT194679-MT194687, MT194690-MT194768, MT194770-MT194772, MT194774-MT194779, MT194781-MT194785, MT194788-MT194790, MT194792-MT194793, MT194796-MT194814, MT194816, MT194818-MT194904, MT194906-MT194908, MT194910-MT194913, MT194915-MT194918, MT194920-MT194956, MT194959-MT194966, MT194968-MT194976, MT194978-MT194997, MT194999-MT195037, MT195039-MT195184, MT195186-MT195189, MT195191-MT195426, MT195428-MT195530, MT195532-MT195533, MT195535, MT347678-MT347681, ON890940-ON890941, ON890948-ON890949, ON890961, ON890964-ON890967, ON890972, ON890986, ON890991-ON890993, ON890997, ON891003, ON891011-ON891013, ON891024-ON891025, ON891043-ON891045, ON891049-ON891052, OQ747368-OQ747574
